# Biased signaling of β2-adrenergic receptor in physiological and pathological states

**DOI:** 10.1186/s11658-026-00976-y

**Published:** 2026-06-12

**Authors:** Yaxin Xu, Vincent Kawuribi, Joseph Adu-Amankwaah, Zheng Gong, Xinrui Li, Yixuan Lei, Zhenyu Wang, Parfait Botolo Sakava, Fengjie Zhao, Xiuhua Pan, Cui Li, Hong Sun, Lu Fu

**Affiliations:** 1 Department of Physiology, School of Basic Medicine, Xuzhou Medical University, Xuzhou, 221004 China; 2 National Demonstration Center for Experimental Basic Medical Science Education, Xuzhou Medical University, Xuzhou, 221004 China; 3 Department of Biomedical Engineering, School of Medical Imaging, Xuzhou Medical University, Xuzhou, 221004 China; 4 Department of Physiology & Biomedical Sciences, Seoul National University College of Medicine, Seoul, 110-799 Republic of Korea; 5 The First Clinical Medicine College, Xuzhou Medical University, Xuzhou, 221004 China

**Keywords:** β2AR, Biased signaling, Health and disease, Precision therapy

## Abstract

The β2-adrenergic receptor (β2AR), a pivotal member of the G protein-coupled receptor (GPCR) family, plays a crucial role in cellular signaling and is extensively involved in many physiological and pathological processes. Unlike the classical β1AR, the β2AR exhibits unique biased signaling properties. Selective activation of specific downstream pathways, such as the β-arrestin pathway, ERK1/2, and PI3K/Akt, is mediated by ligand binding via biased signaling by a specific G protein, resulting in distinct cellular responses. This review provides an in-depth analysis of the complex mechanisms governing biased β2AR-mediated signaling, highlighting its crucial roles in cardiovascular health, respiratory pathologies, neuroregulatory mechanisms, immunological regulation, and tumor biology. Although β2AR-biased signaling is a well-established phenomenon, its underlying mechanisms and pathophysiological implications remain incompletely elucidated compared with traditional signaling modes. This paper summarizes recent studies on the specificity of β2AR signaling and examines its potential therapeutic applications. Future research should concentrate on clarifying the structural foundations of biased signaling, developing biased ligands, and using these discoveries for precision therapeutics. The therapeutic potential of β2AR-biased signaling is being fully explored, which may open new avenues for personalized treatment of various diseases and bring breakthroughs to the field of clinical medicine.

## Introduction

The β2AR is a transmembrane protein belonging to the GPCR superfamily, characterized by seven hydrophobic transmembrane helices [1]. Its primary function is to mediate the signaling of catecholamines (such as adrenaline and noradrenaline) by activating G proteins (mainly Gαs), which increases intracellular cAMP levels and triggers physiological responses [2, 3]. β2AR is widely expressed in various tissues, notably the lungs, heart, kidneys, adipose tissue, skeletal muscle, and smooth muscle [4, 5], where it performs diverse physiological functions. In 1967, Lands and colleagues classified β-adrenergic receptors into β1 and β2 subtypes on the basis of the relative potencies of catecholamines across different tissues, demonstrating that receptors in the heart and adipose tissue (β1) were equally responsive to epinephrine and norepinephrine, whereas those in bronchial and vascular smooth muscle (β2) were significantly more sensitive to epinephrine [6]. β2AR has remained an essential topic of scientific research because it plays a unique role in modulating the effects of catecholamine. The conventional mechanism of β2AR function involves ligand binding to initiate intracellular signaling. However, recent studies reveal the presence of noncanonical signaling pathways. Traditionally, β2AR signaling was thought to rely on the Gαs-mediated cyclic adenosine monophosphate (cAMP) pathway, but emerging evidence highlights its biased signaling properties [7]. Specifically, ligand binding can selectively activate specific downstream pathways (e.g., β-arrestin), eliciting unique cellular responses [8]. In contrast to other receptors, the β2AR, similarly to other class A GPCRs, resides predominantly at the plasma membrane but undergoes constitutive and agonist-stimulated internalization that modulates its signaling output upon ligand binding. Although the expression levels of β2AR differ among different cell types, it is widely distributed in multiple tissues in the body, including the cardiovascular and respiratory system, metabolism-related tissues (such as adipose and skeletal muscle), and the immune and nervous systems [4, 9–11]. The widespread presence of β2AR in these organ systems highlights its profound impact on a wide range of physiological processes. Furthermore, the activity of β2AR is regulated by several mechanisms, including binding to β-arrestin, endocytosis, phosphorylation, and desensitization [12]. These regulatory mechanisms not only modulate receptor function but also play roles in the development and progression of diseases such as asthma, cardiovascular disorders, and certain tumors.

Under normal physiological conditions, β2AR is essential for cardiovascular regulation, as it relaxes vascular smooth muscle to decrease peripheral resistance and regulate blood pressure [12]. In the respiratory system, it dilates bronchial smooth muscle to maintain airway patency [13]. In the nervous system, β2AR controls synaptic plasticity and neurotransmitter release, affecting cognition and mood [14]. In terms of metabolism, it modulates lipolysis and glycogen metabolism, maintaining energy balance [15, 16]. It also affects the immune and reproductive systems by fine-tuning immune cell function [17, 18] and promoting follicle development, ovulation, pregnancy, and the maturation of sperm [19–21]. Under pathological states, the influence of β2AR is amplified, affecting numerous cellular processes such as the cAMP–PKA signaling pathway, calcium signaling, MAPK signaling, PI3K–Akt signaling, cytoskeletal remodeling, inflammatory and immune responses, metabolic regulation, and apoptosis, in addition to activating various kinase signaling cascades. These effects have been validated in our laboratory’s prior studies under multiple stress conditions [22–25].

Notably, β2AR has also been implicated in cancer biology, where it participates in tumor proliferation, invasion, metastasis, and microenvironment remodeling through multiple signaling pathways; these roles are comprehensively discussed in the Cancer section.

Biased signaling, also known as functional selectivity, is a well-known feature in GPCR [26, 27] As a member of the GPCR family, β2AR can selectively activate specific signaling pathways while suppressing others upon ligand binding. For instance, different β2AR agonists may trigger different signaling biases, with some preferentially activating the cAMP–PKA pathway, whereas others may tend to engage unconventional signaling cascades [3, 27–29] This biased signaling phenomenon holds profound implications for receptor pharmacology and drug development. Ongoing research into the molecular mechanisms and pathophysiological effects of β2AR increasingly highlights its potential as a biomarker and therapeutic target for various diseases. However, our current understanding of the precise mechanisms by which β2AR contribute to biased signaling remains incomplete. This review aims to provide an in-depth exploration of β2AR-mediated biased signaling across different physiological systems and pathological conditions. By elucidating its mechanisms and significance in cardiovascular regulation, respiratory function, neurological regulation, immune modulation, and cancer progression, we further emphasize its potential as a versatile and precise therapeutic target with extensive clinical applications.

Critically, biased signaling must be distinguished from canonical, balanced agonism: whereas a balanced agonist activates all receptor-coupled pathways proportionally, a biased agonist preferentially engages one pathway over another. Bias is rigorously quantified using the operational model framework, yielding transduction coefficients log(τ/KA) that are normalized to a reference agonist and compared across pathways as bias factors ΔΔlog(τ/KA); a statistically significant ΔΔlog(τ/KA) ≠ 0 indicates true ligand-directed bias[30]. This quantitative distinction is essential because many apparent signaling differences may arise from cell-state-dependent or system-dependent variations rather than intrinsic ligand bias. Throughout this review, examples in the Cardiovascular System–Cancer sections are explicitly framed as either (i) ligand-directed bias rigorously quantified by ΔΔlog(τ/KA), (ii) functional selectivity (cell-state- or system-dependent effector coupling), or (iii) conventional balanced agonism, in line with the recommendations of Kenakin and Christopoulos [26] and Onaran & Costa [31].

## β2AR signaling transduction

**Fig. 1:**
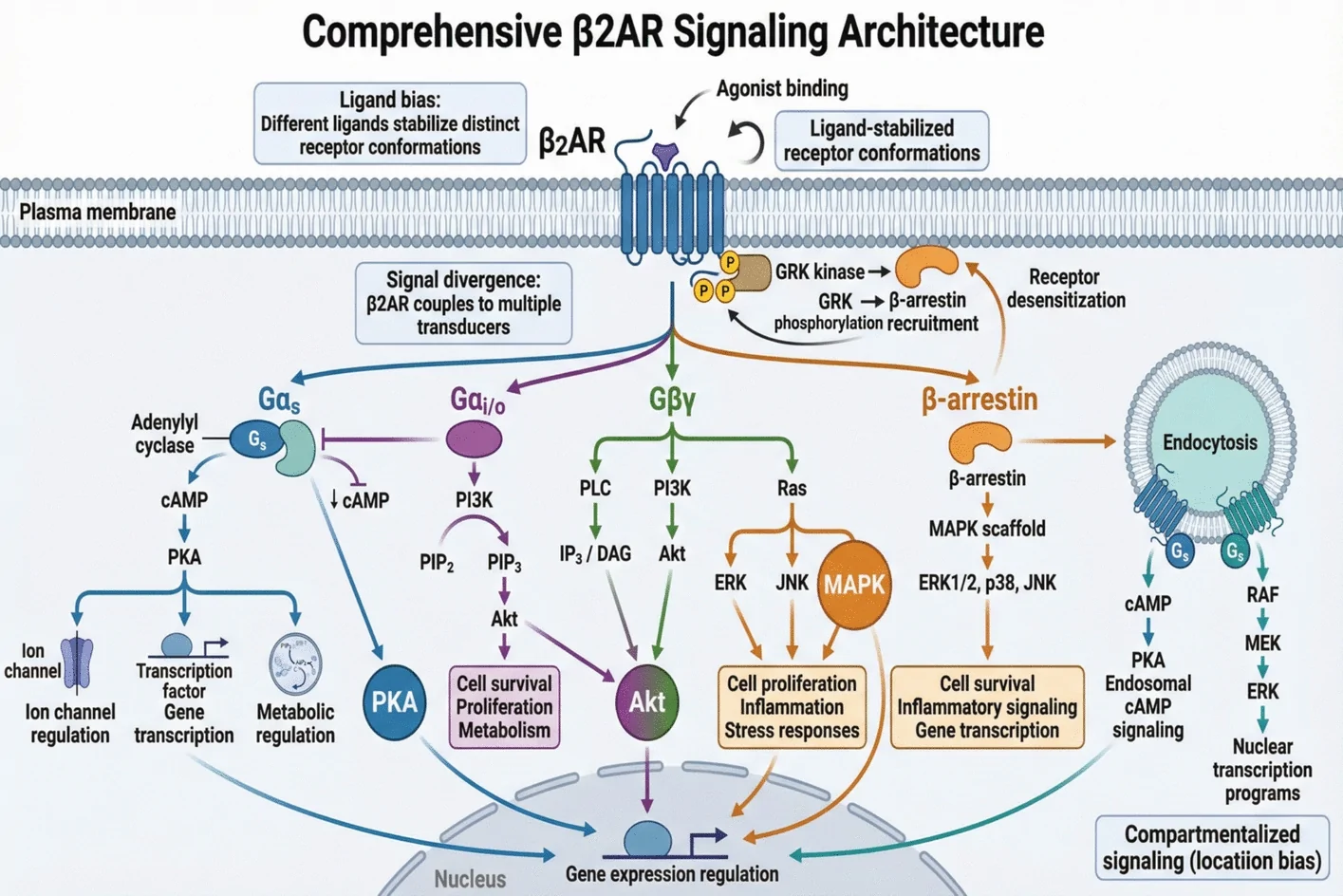
Comprehensive β2-adrenergic receptor (β2AR) signaling architecture. Ligand binding to β2AR at the plasma membrane stabilizes distinct receptor conformations that enable coupling to multiple intracellular transducers, leading to signal divergence and pathway-specific cellular responses. Upon agonist binding, β2AR primarily activates heterotrimeric G proteins. Coupling to Gas stimulates adenylyl cyclase (AC), increasing cyclic AMP (cAMP) production and activating protein kinase A (PKA), which regulates ion channel activity, metabolic processes, and transcriptional responses. In contrast, coupling to Gαi/o inhibits AC activity and reduces intracellular cAMP levels while simultaneously activating phosphoinositide-3-kinase (PI3K), leading to conversion of phosphatidylinositol-4,5-bisphosphate (PIP2) to phosphatidylinositol-3,4,5-trisphosphate (PIP3) and subsequent activation of the Akt signaling pathway involved in cell survival, proliferation, and metabolism. The released Gβγ subunits activate additional signaling cascades including phospholipase C (PLC), which generates IP3 and DAG, and PI3K-dependent pathways that further amplify Akt signaling. Gβγ also activates small GTPases such as Ras, initiating mitogen-activated protein kinase (MAPK) signaling pathways including ERK, JNK, and p38 MAPK, which regulate cell proliferation, inflammatory responses, and cellular stress signaling

The β2AR couples to multiple intracellular transducer proteins, including a variety of proteins, including Gαs, Gαi/o, and Gβγ subunits from the G protein family, as well as β-arrestins. It coordinates complex physiological effects through a sophisticated signaling network whose output depends critically on the conformational state stabilized by the bound ligand. Rather than functioning as a simple binary on/off switch, β2AR exists as a dynamic conformational ensemble in which different ligands stabilize distinct receptor conformations that preferentially engage specific transducer proteins, a concept supported by single-molecule FRET studies [28, 32]. In this process, the activation of G proteins is a core step, while the type of G protein ultimately activated and the selection of downstream signaling pathways are finely regulated by various factors. The binding of the ligand to β2AR is the initial step in initiating signal transduction [33]. Among them, the chemical properties of the ligand play a decisive role. It determines the mainly activated G protein pathway, thus influencing the direction of the entire signal cascade reaction. Ligand binding also induces a conformational change in β2AR. This change is crucial as it can stabilize a specific state of the receptor, making it more likely to interact with Gαs, Gαi/o, Gβγ, and β-arrestin proteins, which is the fundamental for subsequent signal transduction [34–36]. The intracellular environment cannot be ignored either. The expression levels of G proteins and β-arrestin, as well as the presence of various other signaling molecules, will affect the biased activation of downstream effectors [37]. In addition, β2AR has its own unique structural features. For example, it has a structural domain that selectively binds to specific G proteins. These structural domains are similar to precise “molecular switches” that can accurately regulate the interaction between β2AR and different G proteins, ensuring that the signal transduction is both precise and diverse [35, 38]. In conclusion, the biased signaling transduction of β2AR is a complex process determined jointly by ligand properties, receptor conformational changes, the cellular environment, and receptor structural features. These factors work together to coordinate the physiological effects of β2AR, enabling it to make adaptive adjustments according to different physiological and pathological conditions (Fig. 1).

### Gαs signaling

The signaling pathway mediated by the binding of β2AR to Gαs protein is its most classical mode of signal transmission. When a ligand specifically binds to β2AR, the conformation of the receptor changes immediately. This ligand-induced conformational change promotes G-protein coupling by exposing intracellular binding sites, leading to the activation of the coupled Gαs protein. The activated Gαs protein further acts on adenylyl cyclase (AC), enhancing its activity, and subsequently catalyzes the conversion of intracellular ATP into cAMP. As an important second messenger, cAMP can activate PKA. Once PKA is activated, it is capable of phosphorylating a variety of downstream target proteins. These target proteins are widely involved in multiple cellular processes such as ion channel regulation, metabolic process regulation, cell growth, and gene expression [39, 40]. This demonstrates the indispensable and important role of β2AR in regulating various physiological functions of the body.

### Gαi/o signaling

In addition to the traditional Gαs protein pathway, β2AR can also interact with Gαi/o protein. The signaling pathways initiated upon their binding are significantly different from the Gαs pathway. Once Gαi/o protein is activated, it inhibits the activity of AC, resulting in a decrease in the production of cAMP, and the activity of PKA and its downstream signal transduction are also inhibited accordingly [41]. It is worth noting that Gαi/o protein also has the ability to activate PI3K. After PI3K is activated, it catalyzes the conversion of phosphatidylinositol-4,5-bisphosphate (PIP2) into phosphatidylinositol-3,4,5-trisphosphate (PIP3). As a key signaling molecule, PIP3 can bind to and activate serine/threonine kinase (Akt). Once Akt is activated, it further triggers the activation of a series of downstream signaling molecules, participating in the regulation of various key physiological processes such as cell survival, proliferation, and metabolism. This indicates that β2AR plays an important role in regulating cell physiology, especially in aspects related to cell survival and proliferation [42].

### Gβγ signaling

When the β2AR binds to Gβγ, it activates multiple downstream signaling cascades that mediate diverse cellular functions through distinct effector proteins. First, when the β2AR binds to Gβγ, it can activate the PLC–IP3–DAG pathway, which greatly increases the production of IP3 and DAG [36, 43]. Meanwhile, the Gβγ subunit can also activate PI3K, further enhancing the signal intensity of the PI3K–Akt pathway, and playing an important regulatory role in aspects such as cell survival and metabolism [44, 45]. In addition, Gβγ can initiate the mitogen-activated protein kinase (MAPK) pathway by activating small G proteins such as Ras. This signaling cascade includes multiple branches such as extracellular signal-regulated kinase (ERK), c-Jun N-terminal kinase (JNK), and p38 MAPK. Among them, the ERK pathway plays a key role in cell proliferation, differentiation, and survival, while the JNK and p38 MAPK pathways are mainly involved in the regulation processes of cellular stress responses, inflammation, and apoptosis. This demonstrates that β2AR can comprehensively regulate the behavior and fate of cells through its interaction with Gβγ [36, 46].

### β-arrestin signaling

In addition to interacting with various G proteins, the β2AR can also specifically bind to β-arrestin. The signaling pathways triggered by their binding play important roles in multiple aspects of the cell [47]. Following agonist-induced receptor activation, G protein-coupled receptor kinases (GRKs) phosphorylate specific serine and threonine residues on the β2AR C-terminus and intracellular loops. This GRK-mediated phosphorylation creates high-affinity binding sites for β-arrestin, which then sterically hinders further G-protein coupling, thereby attenuating the G protein signaling process. This process of acute signal termination is known as receptor desensitization, which should be distinguished from the subsequent process of receptor internalization (endocytosis). At the same time, as an adaptor protein, β-arrestin can recruit microtubule proteins and other proteins related to internalization, promoting the internalization (endocytosis) of the β2AR. β-arrestin effectively prevents receptor overactivation and protects the cell from damage caused by excessive stimulation through desensitization and internalization mechanisms [48]. β-arrestin also has the function of a scaffold protein, which can activate the MAPK signaling pathway, including important members such as ERK1/2, p38, and JNK [47, 49]. In addition, β-arrestin activates Akt by interacting with PI3K and participates in the regulation of cell survival and proliferation. It can also activate the nuclear factor (NF)-κB signaling pathway by interacting with the IKK complex, playing a role in regulating the inflammatory response and promoting cell survival [50]. Meanwhile, β-arrestin can interact with cytoskeleton-related proteins (such as cofilin and actin) and participate in the remodeling process of the cytoskeleton [51, 52]. The interaction between β2AR and β-arrestin not only regulates the receptor’s activity and intracellular localization, but also influences cellular functions and behavior through multiple mechanisms, providing new insights into the functional versatility of β2AR [53]. Understanding these signaling pathways and their mechanisms of action facilitates the development of more targeted β2AR-targeted drugs for the treatment of related diseases, particularly biased ligands.

The specificity of β-arrestin-mediated signaling is further refined by the “GRK barcode hypothesis,” which posits that distinct GRK isoforms phosphorylate different serine/threonine residues on the β2AR C-terminus, generating unique phosphorylation patterns (“barcodes”) that dictate the conformation and function of recruited β-arrestin. GRK2/3, which are recruited to the receptor in a Gα-dependent manner, primarily promote receptor desensitization and internalization, whereas GRK5/6 phosphorylation drives β-arrestin-dependent signaling through ERK and other effectors [27, 54]. Recent work by Kawakami et al. [55] demonstrated that heterotrimeric Gq proteins act as a molecular switch for GRK5/6 selectivity underlying β-arrestin transducer bias at AT1R and other Gq-coupled receptors (e.g., 5-HT2A, NTS1). For Gs-coupled receptors such as β2AR, however, this Gq-mediated switch does not operate; instead, GRK5/6 selectivity at β2AR is governed primarily by ligand-induced receptor conformational states and the kinetics of direct GRK recruitment to the agonist-occupied receptor [28, 54]. Additionally, the conformational state of the receptor itself is a critical determinant of bias. Using site-specific ^19^F-nuclear magnetic resonance (NMR) probes, Liu et al. [28] showed that distinct ligands stabilize different conformational states of β2AR: agonists primarily shift transmembrane helix VI toward a Gs-coupling conformation, while β-arrestin-biased ligands predominantly affect helix VII conformation. These findings demonstrate that biased signaling arises from structurally distinct receptor conformations rather than a single “active state,” supporting a model in which the receptor exists as a dynamic conformational ensemble that is differentially sampled by biased ligands.

### Compartmentalized β2AR signaling

In addition to signaling from the plasma membrane, β2AR continues to activate G proteins and generate cAMP from endosomal compartments following β-arrestin-mediated internalization. Irannejad et al. [56] demonstrated using conformational biosensors that internalized β2AR maintains an active G protein-coupled conformation on endosomes, producing spatially confined cAMP pools distinct from those generated at the plasma membrane. This “location bias” has important functional consequences: endosome-derived cAMP selectively activates nuclear PKA targets and regulates distinct transcriptional programs. Furthermore, noncanonical β-adrenergic activation of ERK at endosomes has been demonstrated [57], proceeding through an endosomally localized Gs–RAF–MEK axis. Calebiro et al. [58] recently reviewed advances in endomembrane GPCR signaling, highlighting therapeutic implications of targeting receptor signaling at specific subcellular locations. The concept of location bias intersects directly with ligand bias: ligands that promote rapid receptor internalization may preferentially activate endosomal signaling pathways, while ligands that stabilize the receptor at the plasma membrane may bias toward surface-localized cAMP production.

In summary, the biased signaling transduction of the β2AR is collectively determined by ligand properties, receptor conformational changes, cellular environment, and receptor structural characteristics. The detailed pathway descriptions provided above serve as the comprehensive reference for the signaling mechanisms discussed throughout this review; in the organ system-specific sections that follow (Cardiovascular System–Cancer sections), we focus on how these pathways are differentially engaged in specific physiological and pathological contexts rather than redescribing the core signaling cascades. β2AR can interact with a variety of G proteins and β-arrestin. By activating different signaling pathways, the β2AR regulates multiple physiological and pathological processes such as cell proliferation, differentiation, survival, and metabolism.

It is important to note that many examples described above reflect cell-state-dependent or context-dependent signaling variations (e.g., differences in G protein expression levels, GRK isoform availability, or receptor compartmentalization) rather than intrinsic ligand-directed bias. True ligand-directed bias requires demonstration that the same ligand, acting on the same receptor, preferentially activates one pathway over another relative to a reference agonist, as quantified by bias factors. Understanding these mechanisms and distinguishing between these forms of signaling selectivity facilitates the development of precise targeted drugs.

## Roles of β2AR in physiology and pathology

In the following sections, we critically evaluate the evidence for β2AR biased signaling across five major these organ systems. For each system, we assess: (1) whether reported signaling differences represent true ligand-directed bias (as quantified by bias factors ΔΔlog(τ/KA)) or cell-state-dependent/context-dependent variations; (2) the strength of evidence from in vitro versus in vivo models; (3) key controversies and unresolved questions; and (4) translational potential and barriers. This framework allows readers to distinguish robust, reproducible findings from preliminary observations and to identify the most promising avenues for biased ligand development in each therapeutic area.

### Cardiovascular system

β2AR is abundantly expressed in the cardiovascular system, especially abundantly expressed in cardiomyocytes and vascular endothelial cells [59–61]. Under physiological conditions, β2AR modulates the intracellular cAMP signaling pathway by coupling with Gαs and Gαi proteins, influencing physiological processes including cardiac contractility, vasodilation, and cell metabolism. In adult cardiomyocytes, β2AR is predominantly localized in the vesicular structures (such as caveolae) on the cell membrane, coaggregating with Gαi and AC to establish a signaling complex. This compartmentalized distribution endows β2AR signaling with a high level of spatial specificity, enabling precise regulation of the local concentration of cAMP and prevention the cytotoxicity caused by excessive activation of PKA [62]. Studies have shown that in neonatal cardiomyocytes, β2AR mainly enhances contractility through the Gαs–cAMP–PKA pathway, while in adult cardiomyocytes, the positive inotropic effect of β2AR is not accompanied by significant cAMP accumulation, suggesting that there are development-dependent changes in its signaling mechanism [63]. Additionally, in aging, the coupling of β2AR shifts towards biased signaling, and the expression and activation of Gαi increase. Gαi inhibits the activity of adenylyl cyclase and reduces the β2AR/Gαs signal, which in turn leads to a decrease in the cardiac βAR responsiveness in patients with aging and heart failure. In the human atrium, aging increases the inhibition of cAMP signaling mediated by Gαi, which may be one of the important reasons for the decline in cardiac function [64]. Our laboratory’s previous studies have also demonstrated that high concentrations of epinephrine can induce myocardial injury by shifting β2AR coupling from the Gαs to the Gαi signaling pathway, while estrogen supplementation exerts cardioprotective effects by enhancing the activity of the β2AR–Gαs–cAMP–PKA signaling pathway and reducing plasma catecholamine concentrations [23–25, 65, 66]. In addition, β2AR plays a role in cardioprotection via its connections with different signaling pathways. Song et al. elucidated that the heterodimerization of β2AR with serotonin receptor 2B (5-HT2BR) can amplify the Gαi–Akt signaling cascade, thereby exerting antiapoptotic and cardioprotective properties [67]. Fajardo et al. demonstrated that β2AR modulates mitochondrial functionality through interactions with survival kinases εPKC and Akt, thus safeguarding cardiomyocytes against oxidative stress-induced damage [68]. These mechanisms indicate that under physiological conditions, β2AR not only regulates cardiac contractile function but also maintains the survival and metabolic balance of cardiomyocytes through multiple signaling pathways.

Importantly, the functional significance of β2AR in the heart must be understood in the context of β1-adrenergic receptor (β1AR) predominance. In the healthy, nonfailing human myocardium, β1AR and β2AR are expressed at an approximate ratio of 70–80:20–30 in the left ventricle [69]. In heart failure, selective β1AR downregulation shifts this ratio toward approximately 50:50 or even 40:60, substantially increasing the relative contribution of β2AR to total β-adrenergic signaling [70]. This shifting balance is clinically significant because β1AR and β2AR have distinct signaling profiles: β1AR couples predominantly to Gαs and mediates proapoptotic signaling in cardiomyocytes, whereas β2AR exhibits dual coupling to Gαs and Gαi, with the Gαi-dependent arm conferring cardioprotective, antiapoptotic effects via PI3K–Akt [71] Therefore, the relative upregulation of β2AR in failing hearts, combined with its capacity for biased signaling toward cardioprotective pathways, has made it an attractive therapeutic target for biased ligand development.

In the vascular system, β2AR contributes to the relaxation of vascular smooth muscle mainly through the Gαs–cAMP–PKA signaling cascade. This mechanism lowers vascular resistance and consequently improves blood flow [12]. Beyond this pathway, β2AR is also able to activate endothelial nitric oxide synthase (eNOS) via the Gαi–Src–PI3K/Akt route, leading to nitric oxide (NO) production and amplifying vasodilation [72]. In pulmonary arteries, a similar mechanism takes place, where β2AR stimulation engages the same signaling pathway to activate eNOS and elicit NO-dependent vasodilation [73, 74]. In the aorta, it has also been demonstrated that the β2AR-mediated vasodilation function is related to the Gαi signaling pathway [75–77]. The effect of β2AR on blood vessels not only depends on the Gαs signal but is also regulated by Gαi, indicating that β2AR has a dual regulatory mechanism in vasodilation.

However, under pathological conditions, the signaling mechanism of the β2AR undergoes significant changes. These changes lead to a reduction in its physiological protective effects and may even convert it into a driver of pathological damage. Studies have demonstrated that selective activation of the β2AR confers protection against hypoxia- or hydrogen peroxide (H_2_O_2_)-induced apoptosis in neonatal rat cardiomyocytes via activation of the PI3K–Akt signaling pathway. While stimulation of the β1AR can activate MAPK–ERK pathway, its ability to activate PI3K–Akt is weak, with no significant antiapoptotic effect. In contrast, the β2AR-mediated protective effect depends on Gαi proteins and PI3K, and inhibition of the MAPK–ERK pathway does not affect this protective effect. These results indicate that β2AR exerts an antiapoptotic effect in cardiomyocytes through a PI3K-dependent signaling pathway, and this effect is of great significance especially under conditions associated with ischemic stress [78].

In heart failure (HF), the β2AR still binds to Gαs and Gαi proteins, but the activation of Gαi is more prominent. This enhanced Gαi activation further inhibits the activity of AC and the production of cAMP, ultimately leading to a reduction in myocardial contractile function [79]. Furthermore, β2AR coupling to Gαi has been shown to reduce calcium uptake by the sarcoplasmic reticulum through inhibition of the cAMP–PKA pathway, leading to a decrease in calcium handling capacity and further weakening the myocardial contractility. Inhibiting Gαi signaling with pertussis toxin or using agonists that selectively activate the β2AR–Gαs pathway (e.g., fenoterol) can restore the contractile response of β2AR in the failing heart [80, 81]. In contrast, the β-arrestin-dependent signaling pathway promotes the survival of cardiomyocytes. A study demonstrated that ICL1-9, a β-arrestin-biased agonist that selectively activates the β2AR, can enhance cardiomyocyte contractility independently of calcium ions. This finding suggests that targeted therapy against this signaling pathway may represent a potential strategy for improving myocardial function in patients with heart failure [29]. It should be noted that the ICL1-9 pepducin represents a validated example of true ligand-directed β-arrestin bias at β2AR, whereas the Gαs-to-Gαi coupling switch observed in heart failure and aging reflects cell-state-dependent or disease-state-dependent signaling changes rather than intrinsic ligand bias.

In myocardial ischemia/reperfusion (I/R) injury, overexpression of the β2AR exacerbates myocardial damage, resulting in impaired recovery of ATP levels and contractile function. Although inhibition of the Gαi signaling pathway (e.g., using pertussis toxin) can increase basal contractility, it fails to alleviate ischemic injury. This observation indicates that Gαs signaling is the primary contributor to the exacerbation of myocardial damage [82]. Furthermore, β2AR can also exert a cardioprotective effect against myocardial ischemia/reperfusion injury via the Gαi-cPLA2 pathway. When the function of the Gαs–AC pathway is compromised, β2AR tends to activate cPLA2 and exerts a cardioprotective effect through the metabolites of arachidonic acid [83, 84]. Other studies have shown that the β-arrestin-dependent β2AR signaling pathway can reduce mitochondrial superoxide production and activate the RhoA/ROCK pathway. Consequently, this pathway significantly mitigates ischemia/reperfusion injury, reduces infarct size, and improves cardiac function both in vitro and in vivo [85]. Our previous laboratory studies have also demonstrated that under hypoxia/reperfusion conditions, the β2AR maintains the hibernating state of cardiomyocytes via the Gαi–PI3K–Akt pathway, thereby mitigating myocardial damage and cell death [86].

In diabetes-related cardiac disorders, elevated insulin levels inhibit β2AR-mediated cAMP signaling by increasing the expression of phosphodiesterase 4D (PDE4D), thereby contributing to cardiac dysfunction. This process is mediated by the insulin receptor, G protein-coupled receptor kinase 2 (GRK2), and the β-arrestin2-dependent β2AR signaling pathway. Inhibition of β2AR or GRK2 can effectively prevent diabetes-related cardiac dysfunction, indicating that β2AR represents a potential therapeutic target for diabetic cardiomyopathy [87].

In Takotsubo cardiomyopathy, the switching of β2AR signaling transduction is closely associated with acute apical systolic dysfunction. Low concentrations of epinephrine activate the cardiotonic pathway mediated by Gαs protein, whereas high concentrations of epinephrine tend to activate the cardiosuppressive pathway mediated by Gαi protein [88]. This epinephrine-specific β2AR–Gαi signaling pathway may represent an evolutionarily conserved cardioprotective mechanism, which serves to limit catecholamine-induced myocardial toxicity under acute stress conditions. Studies conducted in our laboratory have further demonstrated that under isoprenaline (ISO)-induced stress, β2AR exerts a cardioprotective effect by regulating Gαs/Gαi and its downstream Gαi–PI3K–Akt and Gαi–p38 MAPK signaling pathways. Specifically, this regulation maintains ventricular function, reduces the expression of cardiac injury markers and inflammatory responses, and ameliorates metabolic disorders [23–25, 65, 66].

The β2AR is also involved in cardiac remodeling and fibrosis by regulating gap junctions in cardiomyocytes and the function of cardiac fibroblasts. Excessive activation of β2AR upregulates the expression of connexin 43 (Cx43) in fibroblasts via the PKA signaling pathway and the paracrine action of interleukin-18 (IL-18), thereby promoting the development of myocardial fibrosis and cardiac arrhythmias [89]. In addition, β2AR mediates the expression and secretion of interleukin-6 (IL-6) via the Gαs/ERK1/2 signaling pathway, which promotes the proliferation of cardiac fibroblasts and further exacerbates cardiac fibrosis [90]. These mechanisms indicate that β2AR not only regulates the function of cardiomyocytes in cardiovascular diseases but also participates in the processes of cardiac remodeling and fibrosis by affecting fibroblasts and gap junctions.

Beyond its roles in remodeling and fibrosis, β2AR plays a critical role in postmyocardial infarction cardiac inflammatory repair. β2AR upregulates CCR2 expression via the β-arrestin2–AP-1 signaling axis, promoting leukocyte recruitment to the injured heart. Conversely, β2AR deficiency leads to VCAM-1-mediated leukocyte retention and impaired cardiac repair, ultimately increasing the risk of postinfarction cardiac rupture. This mechanism highlights the critical role of β2AR-biased signaling through β-arrestin2 in regulating the cardiac inflammatory microenvironment [91, 92]

**Fig. 2:**
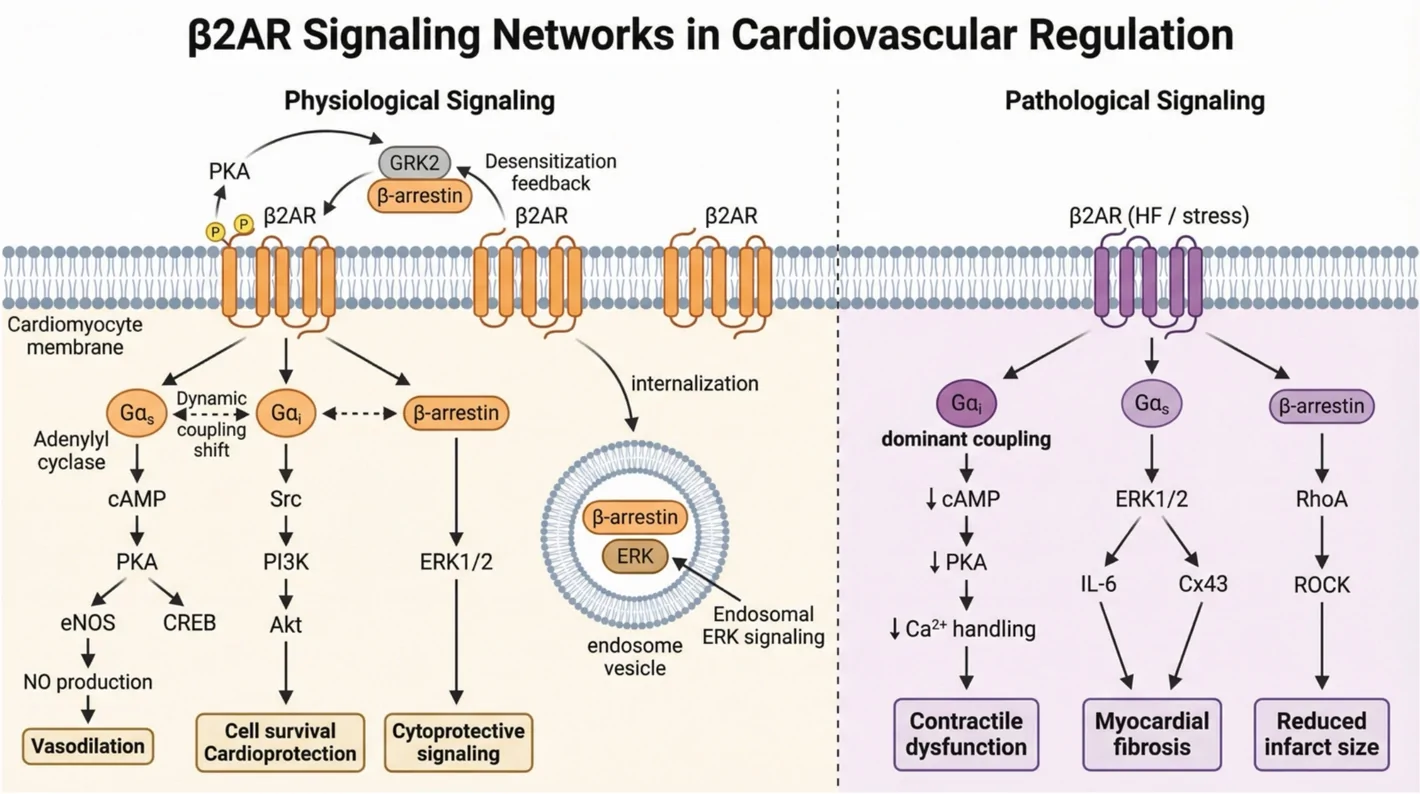
The role of β2AR-mediated signaling in the regulation of cardiovascular system functions. Under physiological conditions, β2AR induces vasodilation via Gαs–cAMP–PKA and Gαi–Src–PI3K–Akt signaling pathways. In neonatal cardiomyocytes, β2AR enhances contraction through Gαs–cAMP–PKA activation. With aging, β2AR shifts toward Gαi activation, which inhibits the cAMP–PKA pathway and leads to cardiac dysfunction. Heterodimerization of β2AR with 5-HT2BR enhances Gαi–Akt signaling, exerting antiapoptotic and cardioprotective effects. Under pathological conditions, such as during ischemia/reperfusion, β2AR activates RhoA/ROCK via β-arrestin, reducing myocardial infarction. In ISO-induced cardiac injury, β2AR regulates Gαs–Gαi binding, inhibits PI3K–Akt, and alleviates inflammation to protect the heart. In H_2_O_2_ and intermittent hypoxia (IH) models, β2AR activates PI3K–Akt, inhibits cardiomyocyte apoptosis, and prevents pulmonary hypertension. In heart failure, β2AR primarily signals through Gαi, inhibits cAMP-PKA, reduces sarcoplasmic reticulum calcium uptake, and impairs contractile function. Chronic excessive activation of β2AR promotes IL-6 secretion via Gαs–ERK1–2 and upregulates Cx43 via PKA, contributing to myocardial fibrosis. Biased signaling examples are indicated: ICL1-9 (pepducin) represents a validated β-arrestin-biased agonist that enhances cardiomyocyte contractility independently of calcium while activating cardioprotective RhoA–ROCK signaling during ischemia/reperfusion. In contrast, the age- and disease-dependent shift from Gαs to Gαi coupling represents cell-state-dependent signaling changes rather than intrinsic ligand bias. Note: pathways depicted as linear for clarity; in vivo, these pathways involve extensive crosstalk, including feedback inhibition of Gαs signaling by GRK2-mediated desensitization, β-arrestin scaffolding of ERK that generates compartmentalized signaling distinct from G protein-mediated ERK activation, and reciprocal regulation between Gαs and Gαi coupling that shifts dynamically with disease progression. Image created with Biorender.com

In patients with hypertension, the signaling transduction of the β2AR also undergoes significant changes. Chronic overstimulation of β2AR amplifies the aortic contractile response to phenylephrine via the Gαi signaling pathway and promotes oxidative stress through eNOS uncoupling, ultimately resulting in endothelial dysfunction [75]. Evidence from studies indicates that in the aorta of mice lacking functional β2AR, the production of superoxide anions by NADPH oxidase is increased, which reduces nitric oxide (NO) bioavailability and intensifies vasoconstrictor activity [76]. In addition, intermittent hypoxia (IH) impairs acute hypoxic pulmonary vasoconstriction (HPV) by activating the β2AR-Gαi mediated PI3K–Akt–eNOS signaling pathway, thereby preventing the development of pulmonary hypertension [74] (Fig. 2).

Overall, these findings delineate the intricate interactions of G protein-coupled signaling pathways. Key limitations of the current cardiovascular literature include: (1) the predominant reliance on rodent models, where βAR subtype ratios and GRK expression patterns differ from humans [69, 70, 93]; (2) the absence of head-to-head comparisons of bias factors for cardiovascular β2AR ligands in native cardiomyocytes; and (3) the challenge of distinguishing ligand-directed bias from the disease-driven Gαs-to-Gαi coupling switch in heart failure. Understanding the mechanisms underlying β2AR’s biased signaling under specific conditions will provide a crucial theoretical foundation for developing personalized therapeutic strategies targeting cardiovascular diseases.

From a translational perspective, the therapeutic exploitation of β2AR biased signaling in the cardiovascular system faces several challenges. Although β-arrestin-biased pepducins such as ICL1-9 have demonstrated cardioprotective effects in preclinical models, their clinical translation remains constrained by an incomplete understanding of system bias versus ligand bias, whereby the same ligand can produce divergent signaling outcomes depending on receptor density, GRK expression patterns, and receptor compartmentalization across different cardiac cell types. Moreover, safety concerns remain significant. β-adrenergic stimulation enhances Ca^2+^ influx through L-type Ca^2+^ channels and increases sarcoplasmic reticulum Ca^2+^ loading; excessive activation may promote Ca^2+^ overload and spontaneous Ca^2+^ release events that can trigger arrhythmias in the failing heart [94]. Current standard-of-care for heart failure relies on β-blockers (e.g., carvedilol, metoprolol), which nonselectively attenuate βAR signaling; the development of biased ligands that selectively preserve cardioprotective β-arrestin signaling while blocking deleterious Gαs-mediated pathways could represent a paradigm shift in heart failure pharmacotherapy. Concretely, β-arrestin-biased β2AR agonists such as ICL1-9 are hypothesized to deliver three measurable clinical advantages over current therapies in heart failure: (i) preserved positive inotropy without the chronic Gαs–cAMP–PKA-driven Ca^2+^ overload that drives ventricular tachyarrhythmias, sudden death, and pump failure progression in patients on chronic nonbiased β-agonist support [80, 94]; (ii) reduced GRK2/β-arrestin-mediated receptor desensitization and tachyphylaxis that limit current β-agonist therapy [95]; and (iii) maintenance or amplification of cardioprotective β-arrestin-dependent RhoA/ROCK and PI3K/Akt signaling that limits infarct size and apoptosis [85]. However, the existing evidence base supports these advantages only at the preclinical level: no β-arrestin-biased β2AR ligand has yet completed a head-to-head clinical trial against standard-of-care β-blockers (carvedilol, metoprolol succinate, bisoprolol), and the cautionary BLAST-AHF experience with the AT1R-biased ligand TRV027, which improved bias-driven end points in vitro but failed to outperform placebo in acute heart failure [96], argues for measured optimism. The clinically validated mortality benefit of carvedilol in HFrEF, attributed in part to its β-arrestin-biased β2AR profile [8, 97, 98], remains the strongest existing clinical signal that β-arrestin-biased β2AR engagement can translate into improved survival, although whether this advantage is mechanistically attributable to β2AR bias as opposed to its dual α1/β1/β2-blockade has been debated.

### Respiratory system

β2AR is widely distributed in various cells of the respiratory system, such as airway smooth muscle cells, alveolar epithelial cells, vascular endothelial cells, and immune cells [99]. Under physiological conditions, β2AR mainly exerts its function by activating G proteins (especially Gαs protein). The activated Gαs protein promotes the activation of adenylyl cyclase (AC), thereby increasing the production of cyclic adenosine monophosphate (cAMP) [100]. Increased cAMP activates protein kinase A (PKA). PKA inhibits myosin light chain kinase, regulates calcium ion exchange, and stimulates Na^+^/K^+^-ATPase. These actions reduce intracellular calcium ion concentration, induce relaxation of airway smooth muscle, and ultimately achieve the function of airway dilation [13, 101, 102]. Additionally, β2AR can directly activate large-conductance calcium-activated potassium (BKCa) channels through the α-subunit of the Gαs protein, further enhancing airway dilation [103, 104]. In alveolar epithelial cells, activation of the β2AR increases the expression of the epithelial sodium channel (ENaC) and the cystic fibrosis transmembrane conductance regulator (CFTR). This promotes the transport of sodium ions and chloride ions, thereby maintaining alveolar fluid balance [105]. Furthermore, β2AR agonists can inhibit the expression of intercellular adhesion molecule-1 (ICAM-1) and the release of granulocyte–macrophage colony-stimulating factor (GM-CSF) in airway epithelial cells through a PKA-dependent mechanism, thereby exerting antiinflammatory effects [106]. Other studies have shown that salbutamol promotes the growth and DNA synthesis of human bronchial epithelial cells (16-HBE and NCI-H292) by activating the cAMP signaling pathway and the MAPK cascade, indicating that the β2AR–cAMP–MAPK pathway plays a key role in regulating airway epithelial cell growth [99]. However, prolonged exposure to β2AR agonists leads to downregulation of β2AR in human airway smooth muscle cells (HASM). This process not only involves receptor internalization and degradation but also increases the expression of miRNA let-7f through the cAMP–PKA–CREB signaling pathway [95].

While quantitative biased agonism studies (e.g., using transduction coefficients or bias factors) remain limited in the respiratory system, the differential G protein coupling documented below illustrates how β2AR can produce functionally selective outcomes through distinct effector arms, providing a foundation for future development of biased ligands targeting respiratory diseases.

Under pathological conditions, alterations in β2AR expression, desensitization, and downstream signaling can impair β2AR-mediated relaxation of airway smooth muscle, thereby unmasking or potentiating procontractile pathways mediated by other receptors such as muscarinic M3 receptors and histamine receptors [94, 95]. Meanwhile, long-term use of β2AR agonists also reduces the density of β2AR, impairs their function, and promotes the release of cytokines IL-6 and IL-8, thereby further exacerbating the inflammatory response [85]. In addition, ICI118,551, an inverse agonist of the β2AR, can reduce the expression of the M3 receptor (M3R) in airway smooth muscle cells through the cAMP signaling pathway, inhibit the production of inositol trisphosphate (IP3), and exert a bronchoprotective effect similar to that of glucocorticoids [107]. In contrast, chronic exposure to long-acting β2AR agonists (LABAs) such as formoterol and salmeterol can promote functional desensitization of β2AR-mediated bronchoprotection in airway smooth muscle through GRK-driven receptor phosphorylation, β-arrestin recruitment, PKA–ERK1/2-dependent induction of phosphodiesterase-4 (PDE4D), and CREB-mediated upregulation of miRNA let-7f, collectively contributing to bronchoprotective tachyphylaxis [95, 108, 109]. When short-acting β2AR agonists (SABAs) are used in combination with long-acting muscarinic antagonists (LAMAs), they can synergistically inhibit muscarinic-induced airway smooth muscle contraction. The mechanism is achieved by reducing protein kinase C (PKC)-mediated calcium ion sensitivity and calcium ion dynamics mediated by KCa channels. This synergistic effect depends on the coupling of Gαs/Gαi proteins, KCa channels, and voltage-dependent calcium channels (VDCs) [110, 111].

Contrary to early assumptions, a substantial body of literature now supports the relevance of biased β2AR signaling in the respiratory system. Wisler et al. [8] established that carvedilol is the prototypical clinically used β-arrestin-biased ligand at β2AR, behaving as an inverse agonist for Gαs–cAMP signaling while simultaneously stimulating β-arrestin-dependent ERK1/2 activation; this dual profile is widely invoked to explain its superior mortality benefit in heart failure relative to nonbiased β-blockers. Subsequent work has elucidated the structural and pharmacological basis of this profile: cryo-EM and biochemical studies show carvedilol stabilizes a distinct β2AR conformation that engages β-arrestin, with its biased efficacy further amplified by positive allosteric modulators of β2AR [97, 112]; however, an independent reanalysis has questioned whether carvedilol per se generates a productive β2AR-β-arrestin signal in some assays [98], underscoring that the magnitude of carvedilol-driven β-arrestin signaling is system-dependent. Hu et al. [108] and Oehme et al. [109] demonstrated that prolonged β-agonist exposure promotes functional desensitization of β2-adrenergic receptor signaling in airway smooth muscle and bronchial epithelial cells, contributing to tachyphylaxis, PKA–ERK1/2-mediated PDE4D induction, and proinflammatory cytokine release that limit sustained bronchodilation. Green et al. [113] identified nonsynonymous β2AR coding polymorphisms (Arg16Gly, Gln27Glu, Thr164Ile) that alter agonist-promoted receptor downregulation and Gαs-coupling efficiency, while a separate ADRB2-linked GRK5 polymorphism [114] modulates agonist-promoted desensitization; together these variants exemplify how genetic variation can shift the balance between Gαs- and β-arrestin-dependent β2AR signaling. Furthermore, Rajagopal et al. [115] provided a quantitative framework for measuring β-agonist bias at β2AR using transduction coefficients, demonstrating measurable bias differences between formoterol, salmeterol, and other clinically used β-agonists. These studies collectively demonstrate that biased signaling at β2AR is not only measurable in the respiratory system but also clinically relevant, particularly in understanding tachyphylaxis, paradoxical bronchoconstriction, and the risks associated with long-acting β-agonist (LABA) monotherapy. Clinically, the relevance of β2AR biased signaling in respiratory medicine is underscored by the well-documented mortality signals associated with long-acting β-agonist (LABA) monotherapy in asthma, which led to Food and Drug Administration (FDA) black box warnings [116]. These adverse outcomes may relate to the chronic β-arrestin-dependent desensitization and proinflammatory signaling induced by sustained β2AR stimulation, distinct from the acute bronchodilatory Gαs–cAMP pathway. The development of G-protein-biased β2AR agonists that maintain bronchodilation while minimizing β-arrestin-mediated tachyphylaxis and inflammation represents an active area of investigation with the potential to improve the safety profile of β-agonist therapy. Recent biased-agonist profiling has explicitly identified Gαs-biased β2AR ligands (ractopamine, dobutamine, higenamine) that activate the Gαs–cAMP–HASM relaxation arm with minimal β-arrestin recruitment and minimal physiological tachyphylaxis in primary human airway smooth-muscle cells [117], a β-arrestin-biased negative allosteric modulator that selectively suppresses β-arrestin-mediated desensitization without blocking Gαs–cAMP signaling [118], and emerging structure–activity relationships linking distinct bias profiles among short- and long-acting β2-adrenoceptor agonists to differential tachyphylaxis risk [119]. Whether such Gαs-biased ligands will translate into measurable clinical benefit, reduced tachyphylaxis, fewer exacerbations, and lower asthma-related mortality compared with current LABAs, remains to be tested in head-to-head trials, and current clinical evidence supports cautious optimism rather than firm advantage.

### Nervous system

β2AR modulates multiple aspects of neural function, including neurotransmitter release, neuronal survival, and synaptic plasticity, through differential engagement of Gαs, Gαi, and β-arrestin signaling arms (see β2AR Signaling Transduction section). Its impact in neurological diseases is particularly significant, involving multiple neurodegenerative diseases and psychiatric disorders, such as Alzheimer’s disease (AD), Parkinson’s disease (PD), anxiety disorders, and depression.

Although systematic quantitative bias analysis has not yet been widely applied in the nervous system, the differential engagement of Gαs, Gαi, and β-arrestin pathways by β2AR in neurons and glial cells demonstrates functional selectivity that has important implications for neurodegenerative disease therapy and biased ligand development.

Under physiological conditions, activation of β2AR enhances synaptic long-term potentiation (LTP) within the hippocampus and dentate gyrus through the Gαs–cAMP–PKA signaling pathway. This mechanism constitutes a fundamental cellular basis for learning and memory [120, 121]. Experimental research has elucidated that the β2AR agonist clenbuterol can stimulate postsynaptic cAMP–PKA signaling and inhibit GABAergic transmission, thus enhancing cognitive performance in the prefrontal cortex [14]. In addition, the β2AR can also promote astrocytes to synthesize brain-derived neurotrophic factor (BDNF) by activating the PKC and cAMP–PKA pathways, thereby regulating neural plasticity [122]. In the motor system, fenoterol, a β2AR agonist, enhances the recruitment and release of synaptic vesicles by switching to a Gαi protein-dependent signaling pathway, improving the function of the neuromuscular junction; its signal transduction depends on the integrity of lipid rafts [9]. In the CA1 region of the hippocampus, β2AR activation can extend the period of associative long-term potentiation (LTP) via the PKA and MAPK signaling pathways, thereby enhancing synaptic plasticity [123–125]. β2AR additionally forms a signaling complex with the GluR1 subunit, Gαs protein, adenylyl cyclase, and PKA. This complex controls the PKA phosphorylation of GluR1, which increases its expression on the postsynaptic membrane and makes the excitatory postsynaptic current (EPSCs) stronger [126, 127]. However, amyloid-β protein (Aβ) can bind to β2AR and induce its endocytosis and degradation. This impairs β2AR-mediated cAMP effects, PKA activity, GluR1 phosphorylation, and AMPA receptor-mediated synaptic currents, thereby damaging adrenergic and glutamatergic activity [128].

Under pathological conditions, β2AR plays a complex and multifaceted role. Research indicates that the accumulation of Aβ is recognized as a hallmark pathological feature of Alzheimer’s disease (AD). Activation of β2AR can enhance the activity of γ-secretase, promote the cleavage of amyloid precursor protein (APP), and increase Aβ production, leading to neuronal damage and synaptic dysfunction [129]. Furthermore, in transgenic murine models of AD, β2AR activation facilitates tau phosphorylation via the PKA-JNK and arrestin-ERK1/2 signaling cascades, which in turn exacerbates neurofibrillary tangles. In contrast, the knockout of β2AR mitigates synaptic damage and improves learning and cognitive function [130]. Other research suggests that β2AR activation may confer neuroprotective effects via antiinflammatory and neurotrophic pathways. For instance, the β2AR agonist clenbuterol hydrochloride has demonstrated considerable antiinflammatory and neurotrophic benefits in a kainic acid (KA)-induced neurodegenerative model, diminishing hippocampus cell apoptosis and decreasing the production of inflammatory factors [131]. Activation of β2AR may increase hippocampus neurogenesis in AD model mice and improve their cognitive deficits [132], whereas inhibition of β2AR exacerbates cognitive impairment in these mice by elevating Aβ levels [133]. The apparently contradictory roles of β2AR in AD likely arise from several sources of experimental variation. First, the ligands used differ in their intrinsic bias profiles: clenbuterol is a partial agonist with potential β-arrestin-biased properties, whereas isoproterenol is a full, relatively unbiased agonist. Second, receptor reserve and expression levels differ between experimental systems (HEK293 overexpression cells versus primary hippocampal neurons versus transgenic mouse models). Third, GRK2 and β-arrestin2 expression varies between cell types and developmental stages. Fourth, dose-dependent switching between Gαs and Gαi coupling may apply in neuronal systems. Fifth, acute versus chronic agonist exposure may yield opposing outcomes due to differential receptor desensitization. Future studies should systematically evaluate these variables using quantitative bias analysis (ΔΔlog(τ/KA)) across matched experimental conditions to resolve these discrepancies.

In Parkinson’s disease (PD), activation of the β2AR can reduce the expression of α-synuclein (SNCA) and alleviate neuroinflammation and oxidative stress responses [134]. In addition, β2AR agonists can further reduce neuroinflammation by inhibiting the activation of immune cells (such as macrophages, glial cells, and T cells) [135]. However, there are contradictions in the research findings regarding the relationship between β2AR agonists and the risk of PD. Some studies indicate that long-term use of β2AR agonists may reduce the risk of PD [136], while others suggest that this apparent protective effect may be confounded by factors such as smoking [137, 138].

**Fig. 3:**
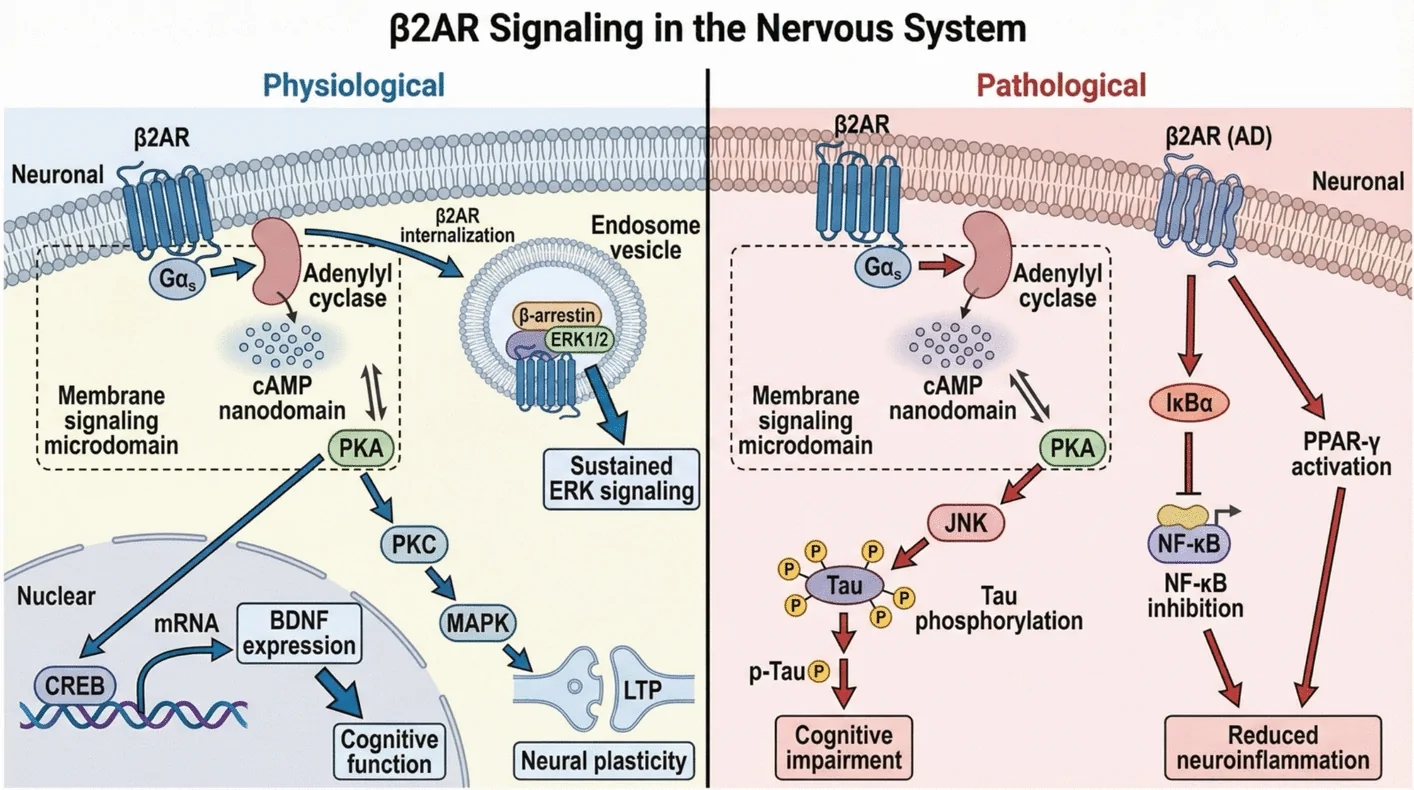
The role of β2AR-mediated signaling in the regulation of nervous system functions. Under physiological conditions, β2AR activates the Gαs–cAMP–PKA signaling pathway to inhibit GABAergic transmission and enhance cognitive function. β2AR promotes brain-derived neurotrophic factor (BDNF) synthesis through PKC and cAMP–PKA pathways, and extends the time window of long-term potentiation (LTP) via the MAPK pathway, thereby enhancing neural plasticity. Under pathological conditions, β2AR facilitates tau protein phosphorylation through PKA-JNK and arrestin-ERK1/2 pathways, leading to cognitive impairment in AD mice. β2AR suppresses the NF-κB pathway by increasing IκBα expression and activates the PPAR-γ pathway, thereby inhibiting the expression of inflammatory factors and attenuating neuroinflammation. Current evidence in the nervous system predominantly reflects conventional β2AR agonism rather than rigorously demonstrated ligand-directed bias; quantitative bias analysis is needed to determine whether the divergent outcomes of β2AR activation in AD (neuroprotective versus Aβ-promoting) arise from differences in ligand bias profiles or from cell-type-specific signaling contexts. Note: neural β2AR signaling involves significant compartmentalization, with distinct cAMP nanodomains generated at the plasma membrane versus endosomes, and feedback regulation through PKA-mediated phosphorylation of the receptor itself, which influences subsequent β-arrestin recruitment and receptor trafficking. Image created with Biorender.com

In anxiety disorders, optogenetic techniques have confirmed that activation of β2AR is sufficient to induce anxiety-like behaviors and reduce social interaction, while specific inhibition of β2AR produces anxiolytic-like behaviors and promotes social interaction [139]. In addition, the regulatory role of β2AR in neuroinflammation is mainly achieved by inhibiting the NF-κB pathway and activating PPAR-γ [140, 141]. β2AR agonists, such as clenbuterol, can inhibit the activity of NF-κB by increasing the expression of IκBα and reducing the release of proinflammatory factors [142], while simultaneously promoting the expression of antiinflammatory molecules and enhancing the antiinflammatory effects of glucocorticoids [143] (Fig. 3).

Although the current research on the biased signaling of β2AR in the nervous system is not sufficient, its complex roles under physiological and pathological conditions have been widely confirmed. A major controversy in this field concerns whether β2AR activation is neuroprotective or neurotoxic in Alzheimer’s disease, with conflicting evidence likely arising from differences in agonist selection (partial versus full agonists, biased versus balanced ligands), dosing regimens, disease stage, and regional receptor expression [129, 130, 132, 133, 141]. Furthermore, no study has yet applied quantitative bias analysis to neurological β2AR agonists to determine whether observed differences reflect intrinsic ligand bias or tissue-specific signaling contexts. Further investigation of the biased-signaling mechanisms of β2AR in the nervous system is therefore warranted. Notably, most examples described in this section reflect conventional agonism or cell-type-dependent signaling variations (e.g., the neuroprotective effects of clenbuterol, the context-dependent roles in AD) rather than rigorously demonstrated ligand-directed bias. Future studies should employ quantitative bias analysis (ΔΔlog(τ/KA)) with reference agonists to determine whether the divergent outcomes reported for β2AR agonists in the nervous system arise from intrinsic ligand bias or from tissue-specific differences in receptor expression, GRK availability, and effector coupling. To make the trichotomy explicit per example: examples representing conventional agonism include β2AR enhancement of LTP via Gαs–cAMP–PKA [120, 121], BDNF synthesis in astrocytes [122], LTP at CA1 [123–125], and the GluR1 signaling complex [127]; examples representing functional selectivity/system bias include the divergent neuroprotective versus neurotoxic outcomes of β2AR agonists in AD models [129, 130, 132, 133, 141], the Gαi-dependent switch driving fenoterol-enhanced neuromuscular transmission [9], and the dose- and context-dependent PD outcomes [134–138]; the only example in the nervous system that approaches rigorously demonstrated ligand-directed bias is the partial-agonist clenbuterol relative to a balanced reference such as isoproterenol, and even there ΔΔlog(τ/KA) measurements have not been reported.

### Immune system

β2AR is crucial for immune control, encompassing several molecular processes that differ among immune cell types and disease contexts. Under normal physiological conditions, β2AR maintains immune homeostasis by regulating the functions of immune cells and preserving immune tolerance. For example, in T cells, activation of this receptor encourages the release of Th2 cytokines, including IL-4 and IL-5, while suppressing the production of Th1 cytokines such as IFN-γ through the cAMP–PKA signaling route. It also inhibits the activity of calcineurin (CaN) downstream of the T cell receptor (TCR). This process relies on the interaction between PKA and A-kinase anchoring proteins (AKAPs), thereby reducing the production of IL-2 and IFN-γ, inhibiting T cell proliferation, and ultimately influencing the bias of the immune response [91, 144, 145]. It is worth noting that the β2AR signaling also plays a key role in regulating Th1/Th2 balance by inhibiting the activation of NF-κB and AP-1. This is manifested by downregulating the secretion of Th1-type cytokines (such as IFN-γ) while promoting the production of Th2-type cytokines (such as IL-4 and IL-5) [146]. For the Th17 cell subset, β2AR shows bidirectional regulatory characteristics: the agonist terbutaline enhances the secretion of IL-17A in memory Th17 cells. It upregulates the expression of RORC through the cAMP–PKA pathway. In contrast, the biased agonist nebivolol selectively inhibits the secretion of IL-17A through a nonclassical signaling pathway, and at the same time downregulates the phosphorylation of RORC and NF-κB p65 [147]. In B cells, β2AR agonists inhibit B cell proliferation and antibody secretion [10, 11]. In macrophages, the regulation of β2AR shows bidirectionality. It can inhibit NF-κB through the cAMP–PKA pathway to exhibit an antiinflammatory effect [148, 149], and it can also enhance the production of IL-1β and IL-6 through the ERK–p38–ATF axis, showing a proinflammatory effect [17]. This bidirectional regulation likely arises from the differential coupling of β2AR to distinct G proteins (such as Gαs and Gαi) and the crosstalk among associated signaling cascades. For instance, the reciprocal regulation between β2AR and the TLR4 signaling pathway can suppress the synthesis of proinflammatory mediators [18, 150], and under certain circumstances, β2AR can facilitate M2 polarization of macrophages through the PI3K–Akt pathway, exacerbating fibrosis [151, 152]. In addition, β2AR inhibits the activation of autoreactive T cells by regulating the function of dendritic cells (DCs) to prevent autoimmune reactions [11, 153]. The bidirectional immunomodulatory effects of β2AR promoting either proinflammatory or antiinflammatory programs depending on cell type, activation state, and downstream effector coupling, represent a clear example of context-dependent functional selectivity rather than a single ligand-directed bias profile, and are directly relevant to biased-ligand development for autoimmune and inflammatory disease.

**Fig. 4:**
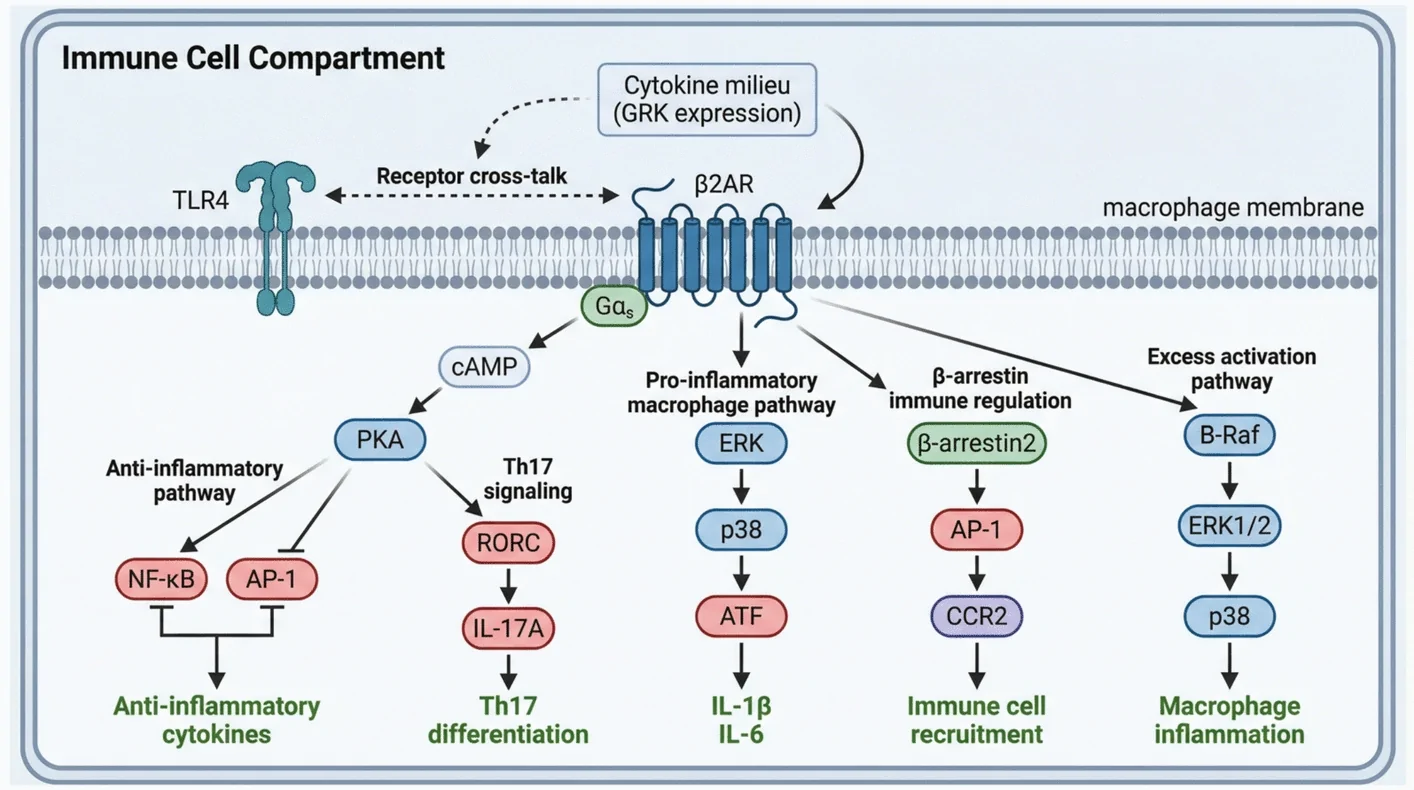
The role of β2AR-mediated signaling pathway in the regulation of immune system functions. Under normal physiological conditions, β2AR inhibits inflammation by activating the cAMP–PKA pathway and suppressing NF-κB and AP-1 signaling in T cells. In Th17 cells, β2AR exerts dual effects: it increases IL-17A secretion and upregulates RORC via cAMP–PKA, while also selectively inhibiting IL-17A and downregulating RORC and NF-κB to exert antiinflammatory effects. In macrophages, β2AR has dual functions: it exerts antiinflammatory effects by inhibiting NF-κB via cAMP–PKA, but promotes the production of IL-1β and IL-6 via the ERK–p38–ATF axis to exert proinflammatory effects. Under pathological conditions, β2AR upregulates CCR2 via the β-arrestin2–AP-1 axis to inhibit inflammation after myocardial infarction. β2AR upregulates Trem1 via the cAMP–PKA–CREB pathway, amplifies LPS-induced inflammation, and delays hindlimb wound healing in mice. β2AR inhibits the production of TNF-α, IL-18, and IL-6 to suppress neuroinflammation via PI3K and p38 MAPK signaling pathway. Activation of β2AR leads to PKA phosphorylation, downregulates TLR4, and inhibits LPS-induced TNFα production, thereby suppressing macrophage inflammation. However, excessive β2AR activation induces macrophage inflammation via the B-Raf–ERK1/2 and p38 pathways. The dual proinflammatory and antiinflammatory outcomes of β2AR activation in immune cells may reflect context-dependent engagement of different signaling arms rather than intrinsic ligand bias; however, the nebivolol-mediated selective inhibition of Th17 differentiation (Hajiaghayi et al.) represents an example of pharmacologically distinct β-arrestin-biased agonism with therapeutic relevance for autoimmune diseases. Note: immune cell β2AR signaling is subject to allosteric regulation by the local cytokine milieu, receptor cross-talk with costimulatory receptors (e.g., TCR, TLR), and cell-activation-state-dependent changes in GRK–β-arrestin expression that fundamentally alter the signaling output from the same receptor-ligand interaction. Image created with Biorender.com

Under pathological conditions, β2AR exhibits complex bidirectional regulatory effects, participating in both protective responses and exacerbating disease progression. This functional diversity stems from differential activation of its signaling pathways and cell-specific modulation of immune cells. The role of β2AR in cardiac inflammatory repair (discussed in detail in Cardiovascular system section) further illustrates the tissue-specific immunomodulatory functions of this receptor [91, 92]. Regarding antiinflammatory effects, β2AR activation primarily exerts immunomodulatory functions via the cAMP/PKA signaling pathway. Studies have shown that the activation of β2AR induces PKA phosphorylation, which in turn downregulates the expression of Toll-like receptor 4 (TLR4) and inhibits lipopolysaccharide (LPS)-induced production of tumor necrosis factor α (TNFα) [150]. In experiments using porcine macrophages and human peripheral blood mononuclear cells (PBMCs), β2AR stimulation significantly suppressed LPS-induced secretion of TNFα and IL-8, while promoting the production of the antiinflammatory cytokine IL-10, without affecting antibacterial function. This effectively prevents the polarization of macrophages toward a proinflammatory phenotype [154, 155]. Notably, the long-acting β2AR agonist salmeterol can inhibit the production of proinflammatory M1 mediators (e.g., TNF-α, IL-18, IL-6, chemokines, and reactive oxygen species) in LPS-activated microglia by activating multiple signaling pathways, including cAMP–PKA–CREB, PI3K, and p38 MAPK. Simultaneously, it enhances the secretion of the antiinflammatory M2 cytokine IL-10, and promotes microglial transition from a proinflammatory M1 to an antiinflammatory M2 phenotype. This provides new insights for treating neuroinflammatory diseases [156]. However, β2AR can also exhibit proinflammatory properties under certain conditions. For instance, in the absence of proinflammatory stimuli, β2AR can induce macrophage production of IL-1β and IL-6 via the B-Raf–ERK1/2 and p38 pathways (independent of PKA or NF-κB) [17]. Additionally, β2AR agonists upregulate the expression of Trem1 in macrophages via the cAMP–PKA–CREB pathway, thereby enhancing lipopolysaccharide (LPS)-induced inflammatory responses and delaying wound healing in the hindlimbs of mice [157]. In addition, in terms of adaptive immune regulation, β2AR also has a more complex regulatory network. In the collagen-induced arthritis (CIA) model, norepinephrine inhibits the Th17 differentiation of CD4^+^ T cells through the β2AR–PKA pathway [144]. Regarding regulatory T cells (Tregs), β2AR activation enhances the phosphorylation of CREB and the expression of CTLA-4 via the cAMP–PKA signaling pathway. This mechanism not only strengthens the suppressive function of Tregs but also promotes the conversion of Foxp3-negative cells into induced Tregs [11] (Fig. 4). Notably, a study on patients with multiple sclerosis (MS) has revealed the dual regulatory mechanism of norepinephrine on CD4^+^ T cells: it can simultaneously inhibit the secretion of IL-17 and IFN-γ, and there is a unique phenomenon: β2AR antagonists enhance the inhibitory effect on IL-17 but weaken the inhibitory effect on IFN-γ. Further experiments have confirmed that β2AR blockade itself can selectively inhibit the production of IL-17, while the agonist form, tromidol, can specifically reduce the level of IFN-γ. This finding provides a new perspective for understanding the neuroimmune regulatory mechanism of multiple sclerosis [158]. These research findings collectively reveal the important mechanism by which β2AR dynamically regulate T-cell function through interactions via multiple pathways. They provide new theoretical bases and potential intervention strategies for targeted therapy of autoimmune diseases and inflammatory diseases.

Although significant progress has been made in understanding the role of β2AR in immune regulation, the biased signaling mechanism underlying their immunomodulatory effects remains poorly understood. A fundamental unresolved question is whether the bidirectional immunomodulatory effects of β2AR (proinflammatory in some contexts, antiinflammatory in others) arise from true ligand-directed bias at the receptor level or from cell-type-specific differences in downstream effector expression (e.g., varying GRK/β-arrestin ratios across immune cell subsets). Additionally, most immunological studies use nonselective β-agonists (isoproterenol, norepinephrine), making it difficult to attribute effects specifically to β2AR versus β1AR or β3AR. Filling this knowledge gap is crucial for fully exploiting the therapeutic potential of β2AR in immune-related diseases.

### Cancer

Recent studies have found that the β2AR is highly upregulated in a variety of cancers, including breast cancer, hepatocellular carcinoma, pancreatic cancer, and prostate cancer. It participates in the processes of tumor proliferation, invasion, metastasis, and angiogenesis through multiple signaling pathways.

In pancreatic cancer, chronic stress can activate the β2AR–HIF–1α axis by increasing the level of adrenaline, upregulating the expressions of MMP-2, MMP-9, and VEGF, thus promoting the growth and angiogenesis of pancreatic cancer [159]. The β2AR antagonist ICI118,551 can be used in combination with propranolol to reduce the invasive and proliferative capabilities of pancreatic cancer cells by dual inhibition of the cAMP–PKA and Ras–MAPK pathways. Its mechanism of action includes downregulating the activity of transcription factors NF-κB, AP-1, and CREB, as well as decreasing the expression of VEGF, COX-2, and MMP-2/9 [160, 161]. In addition, β2AR interacts with aldehyde-ketone reductase family 1 member B1 (AKR1B1) in pancreatic cancer and jointly activates the ERK1/2 signaling pathway. This interaction promotes the proliferation of cancer cells in the S phase and inhibits early apoptosis [162].

In gastric cancer, the β2AR agonist salbutamol facilitates epithelial-mesenchymal transition (EMT), migration, and invasion through ERK phosphorylation [163]. β2AR also promotes the JAK–STAT3 signaling pathway to enhance epithelial-mesenchymal transition (EMT) by upregulating PlexinA1 expression [164]. Sympathetic nerve infiltration enhances the proliferation, migration, and invasion of gastric cancer cells via the STAT3–ERK–YKL-40 signaling pathway, and induces M2 macrophage infiltration and angiogenesis [165]. Also, the β2AR agonist isoproterenol makes gastric cancer cells release VEGF, increases the levels of plexin-A1 and VEGFR2, and creates a positive feedback loop that encourages angiogenesis [166]. Chronic stress activates the β2AR–cAMP–CREB–AMPK–ULK1 signaling pathway, which leads to autophagy that promotes tumors and speeds up the growth of gastric cancer [167, 168].

In breast cancer, the functional role of β2AR is signaling is multifaceted and context-dependent. Research indicates that a low concentration of the β2AR agonist ISO enhances breast cell adhesion via the Gαs–cAMP–Epac pathway, whereas a high concentration of ISO inhibits Erk1/2 phosphorylation-dependent proliferation through the Gαs–cAMP–PKA pathway. This demonstrates the dose-dependent biphasic regulation of β2AR signaling [3]. In triple-negative breast cancer (TNBC), the NE–β2AR axis not only directly promotes proliferation but also drives sympathetic nerve regeneration by inducing the secretion of nerve growth factor (NGF), thereby forming tumor-promoting positive feedback [169]. Additionally, activation of the β2AR pathway activates NF-κB to induce the production of interleukin-6 (IL-6), which then activates the STAT3 signaling pathway in macrophages in a paracrine manner and induces the polarization of macrophages toward the M2 type, thereby promoting breast cancer metastasis [170]. In Her2-positive breast cancer, the β2AR promotes the activation of the Her2–STAT3-mediated PI3K–Akt–mTOR pathway by upregulating miR-21–MUC-1 and inhibiting miR-199a–b-3p, leading to trastuzumab resistance. In contrast, β-blockers can reverse this effect and improve patient survival rates [171]. In addition, the β2AR agonist can significantly reduce the baseline phosphorylation level of ERK1/2 in triple-negative breast cancer (TNBC) cell lines MDA-MB-231 and MDA-MB-468. Its mechanism of action lies in upregulating the expression of the dual-specificity phosphatase DUSP1 and enhancing the activity of protein phosphatase PP1, which indicates that its signal regulation process is quite complex [172]. Preclinical studies have confirmed that the β-blockers propranolol and ICI 118,551 can significantly inhibit the viability of triple-negative breast cancer (TNBC) cells, induce cell cycle arrest, and trigger cell apoptosis by downregulating the ERK–COX-2 signaling pathway [173]. Chronic stress inhibits PPARγ via the β2AR–AC, promoting VEGF–FGF2-mediated angiogenesis in breast cancer, and this process can be blocked by β2AR antagonists [174]. These findings systematically reveal the molecular network through which β2AR regulate breast cancer proliferation, metastasis, drug resistance, and microenvironment remodeling via multiple pathways (e.g., cAMP–PKA–STAT3, ERK, and NF-κB–IL-6). In the tumor microenvironment, norepinephrine (NE) activates STAT3 via the β2AR–PKA, promoting the invasion of ovarian cancer cells and the secretion of MMP-2/9. This process is independent of IL-6 but can be blocked by propranolol or PKA inhibitors [175].

In prostate cancer, the β2AR agonist isoproterenol upregulates the expressions of target genes such as c-Myc, Cyclin D1, and VEGFA by activating the Shh signaling pathway, promoting the proliferation of tumor cells [176]. The β2AR also forms a complex with c-Src via the scaffold protein β-arrestin2, consequently activating the ERK1/2 signaling pathway to promote the proliferation and migration of prostate cancer cells [177]. In addition, the inhibition of β2AR can enhance the sensitivity of castration-resistant prostate cancer to docetaxel by activating the caspase apoptotic pathway and the Atg5–AMPK–mTOR autophagic pathway. Among these mechanisms, β2AR mediates the crosstalk between apoptosis and autophagy by regulating the p38 MAPK and JNK–c-Jun–FOXO3a signaling pathways [178]. More interestingly, inhibition of β2AR can also promote necroptosis in prostate cancer cells, which is characterized by increased expression levels of key proteins in the RIP–MLKL pathway. Experiments have confirmed that there is a direct interaction between β2AR and RIP. Combination therapy using β2AR inhibitors and necroptosis inhibitors can more effectively inhibit tumor proliferation [179].

Additionally, in hepatocellular carcinoma (HCC), the activation of β2AR in hepatic stellate cells (HSCs) promotes the proliferation and migration of HCC cells through the Akt signaling pathway, while the overexpression of β-arrestin2 can inhibit this process. The β2AR agonist terbutaline promotes the progression of liver cancer by downregulating the expression of GRK2 in M2-type tumor-associated macrophages (TAMs) and activating the cAMP–PKA–CREB and cAMP–IL-6–STAT3 signaling pathways [180]. In colorectal cancer (CRC), the β2AR antagonist inhibits the viability of cancer cells and induces G1-phase cell cycle arrest by blocking the EGFR-Akt–ERK1/2 pathway transactivated by β2AR [181]. In clear cell renal cell carcinoma (ccRCC), the blockade of β2AR demonstrates a significant antitumor effect by impeding the HIF-2α–CAIX–VEGF signaling axis as well as the nuclear translocation of NF-κB–p65 [182, 183]. In neuroblastoma (NB), the stimulation of β2AR activates the autophagy pathway via the CREB-mediated upregulation of ULK1, thereby enhancing the proliferation of NB cells [184]. Similarly, in lung adenocarcinoma, activation of β2AR has been shown to drive tumor progression by elevating MMP-2, MMP-9, and VEGF levels through the ERK1/2–CREB pathway [185] (Table 1).

**Table 1:** The mechanisms of β2AR in cancer

Model/disease	Cells/tissue	β2AR	Mechanism	Effects	Ref.
Pancreatic cancer	MIA PaCa-2 and BxPC-3	activated	activates the β2AR–HIF-1α axis, upregulating the expressions of MMP-2, MMP-9, and VEGF	promotes the growth and angiogenesis	[159]
	MIA PaCa-2 and BxPC-3, PanCa	inhibited (with propranolol)	inhibition of the cAMP–PKA and Ras–MAPK pathways, downregulation of the activities of NF-κB, AP-1, and CREB, VEGF, COX-2, and MMP-2/9	reduces the invasion and proliferation	[160, 161]
	CFPAC-1 and PANC-1	activated (with aldehydeketone)	activates the ERK1/2 signaling pathway	promotes the proliferation of cancer cells in the S phase and inhibiting early apoptosis	[162]
gastric cancer	tumor tissue from gastric cancer mice MGC803 and SGC-7901	activated	induces ERK phosphorylation, activates the JAK-STAT3	promotes migration and invasion	[162, 164]
	tumor tissues from patients with gastric cancer AGS and SGC-7901	activated	activates the STAT3–ERK-YKL-40 signaling pathway	promotes proliferation, migration, and invasion	[165]
	tumor tissues from patients with gastric cancer HUVEC, MGC803, and HGC27	activated	secretes VEGF, upregulates the expressions of plexin-A1 and VEGFR2, forms a positive feedback loop	promotes angiogenesis	[166]
	tumor tissues from patients with gastric cancer SGC-7901 and BGC-823 GES-1, BGC823, HGC27, MKN45, SGC7901, AGS, and MGC803	activated	activates cAMP–CREB–AMPK–ULK1 signaling axis	induces autophagy, accelerating the progression of gastric cancer	[167, 168]
Breast cancer	MCF-10A	activated (a low concentration ISO)	activates the Gαs–cAMP–Epac pathway	enhances the adhesion of breast cells	[3]
		activated (a high concentration of ISO)	activates the Gαs–cAMP–PKA pathway	inhibits proliferation	
	MDA-MB-231	activated	activates NF-κB to induce IL-6, activates the STAT3 signaling of macrophages to induce the M2 polarization of macrophages	promotes the metastasis	[170]
	MDA-453, BT474, MCF-7/Her2	activated	upregulates miR-21–MUC-1 and inhibiting miR-199a/b-3p, activates PI3K–Akt–mTOR pathway	leading to trastuzumab resistance	[171]
	MDA-MB-231	inhibited	downregulates the ERK–COX-2 signaling pathway	induces cell cycle arrest, and apoptosis	[173]
Ovarian cancer	SKOV3	activated	activates STAT3 through β2AR–PKA	promotes the invasion	[175]
Prostate cancer	PCa cell lines PC‐3 and LNCaP	activated	upregulates the expressions of c-Myc, Cyclin D1, and VEGFA by activating the Shh signaling pathway	promotes the proliferation	[176]
	PC-3 and 22Rv1	activated	regulates the p38 MAPK and JNK–c-Jun–FOXO3a signaling pathways	mediates apoptosis and autophagy	[178]
Liver cancer	Tumor tissues from patients with liver cancer LX-2, HepG2, and SMMC-7721	activated	activates the cAMP–PKA–CREB and cAMP–IL-6–STAT3 signaling pathways	promotes the progression	[180]
Clear cell renal cell carcinoma	primary VHL-ccRCC, and 786-O cells	inhibited	inhibites the HIF-2α–CAIX–VEGF signaling axis and the nuclear translocation of NFĸB–p65	antitumor	[182, 183]
Lung adenocarcinoma	A549 cells	activated	upregulates the expressions of MMP-2/9 and VEGF through the ERK1/2/CREB signaling pathway	promotes the progression	[185]

Overall, β2AR contributes to cancer development and spread through a variety of interconnected signaling routes that differ among tumor types. A critical gap in the cancer literature is the almost complete absence of studies examining biased signaling per se: the vast majority of studies employ nonselective β-agonists or antagonists and measure downstream phenotypic outcomes (proliferation, migration, apoptosis) without quantifying pathway-specific bias. Moreover, the tumor microenvironment introduces confounding variables including variable receptor density, hypoxia-induced receptor modifications, and paracrine catecholamine release that complicate interpretation. While current research highlights the vital importance of β2AR-biased signaling, numerous mechanistic details remain to be elucidated. Ongoing investigation into these pathways will be critical for the development of selective β2AR-targeted therapies that can enhance precision oncology. However, it is important to recognize that the majority of cancer studies described above demonstrate conventional β2AR agonism or antagonism through established G protein pathways (e.g., cAMP–PKA, ERK–MAPK, PI3K–Akt) rather than true ligand-directed biased signaling. The application of quantitative bias analysis to cancer models, particularly distinguishing G protein-biased from β-arrestin-biased responses in tumor microenvironments, remains an important unmet need. Specifically, in the trichotomy framework: virtually all cancer-section examples (pancreatic, gastric, breast, prostate, hepatocellular, colorectal, ccRCC, neuroblastoma, lung adenocarcinoma; [159–164, 166–170, 172–174, 176, 178–181, 183–185]) are conventional β2AR agonism or antagonism measured by downstream phenotypic outcomes (proliferation, migration, apoptosis); the dose-dependent biphasic ISO response in MCF-10A breast cells [3] represents functional selectivity/system-bias rather than ligand-directed bias; and no cancer study to date has reported quantified ΔΔlog(τ/KA) bias factors at β2AR. Application of the operational model framework to tumor-microenvironment β2AR signaling, particularly with paired G-protein versus β-arrestin readouts in matched tumor-cell systems therefore remains the most important methodological gap for translating biased β2AR pharmacology into precision oncology.

## Pharmacology and drug development

Substantial progress has been made in understanding the pharmacology and therapeutic development of β2AR. Ligands acting on β2AR are commonly divided into two principal classes: natural and synthetic. Natural ligands such as adrenaline and norepinephrine function as endogenous agonists that regulate key physiological processes, including bronchodilation and cardiac activity through their canonical signaling pathways [2, 4]. While system-dependent signaling differences exist (e.g., preferential Gαs coupling in bronchial smooth muscle versus dual Gαs/Gαi coupling in cardiomyocytes), it is widely accepted that endogenous catecholamines are balanced, nonbiased agonists at β2AR and do not exhibit the ligand-directed bias characteristic of purpose-designed biased agonists such as carvedilol or ICL1-9. In contrast, synthetic ligands display higher receptor selectivity. Among them, short-acting agonists (e.g., salbutamol) and long-acting agonists (e.g., formoterol and salmeterol) are widely employed in the treatment of asthma and chronic obstructive pulmonary disease (COPD). These agents promote bronchodilation primarily by activating the Gαs–cAMP signaling cascade; however, prolonged administration can result in receptor desensitization and downregulation [99, 108, 186]. In recent years, the development of biased ligands has become an important direction. For example, the compound TRV027 can selectively activate the Gαs–cAMP pathway while reducing the β-arrestin-mediated internalization and inactivation processes, thereby optimizing therapeutic effects and reducing side effects [187–189]. In addition, inverse agonists (such as ICI-118551) can inhibit the basal activity of β2AR, and allosteric modulators can finely regulate the receptor function through allosteric sites, providing new strategies for precise intervention under pathological conditions [190, 191]. In disease treatment, β2AR targeted drugs are not only used for respiratory diseases but also show potential in heart failure, immune regulation, and the nervous system [80, 110, 135, 147]. For instance, β-arrestin biased agonists (such as ICL1-9) provide new approach for the treatment of heart failure, as they can activate cardioprotective pathways [29] (Table 2).

**Table 2:** The pharmacology and drug development of β2AR in different systems

Strategy/ Drug	Animal/ Cell	Model/disease	β2AR	Mechanism	Effects	Ref.
Cardiovascular system						
Zinterol (ZINT)	Neonatal ventricular myocytes	Apoptosis induced by hypoxia or H2O2	Activated	Increases the activation of MAPK–ERK, the activity of PI3K and the phosphorylation of Akt–PKB	Inhibits cell apoptosis	[78]
ICI-118,551	Rabbit	Heart failure	Inhibited	Inhibits sarcoplasmic reticulum Ca^2+^ overload	Improves arrhythmia	[80]
ICL1–9	HEK293 cells, adult murine cardiac myocytes	Congestive heart failure	Activated	Promotes β2AR phosphorylation, β-arrestin recruitment, β2AR internalization, and β-arrestin–biased signaling	Stimulates cardioprotective signaling and inotropic effects	[29]
ICL1-9	Mice	Ischemia/reperfusion	Activated	Reduces mitochondrial superoxide production and activates the RhoA/ROCK pathway	Reduces infarct size, cardiomyocyte death, and improved cardiac function	[85]
Carvedilol	Mice	Type 2 diabetes mellitus	Inhibited	Attenuates phosphorylation of extracellular regulated protein kinase and PDE4D induction	Prevents contractile dysfunction	[87]
Respiratory system						
Formoterol	ASMCs	ACh induces cell contraction	Activated	Activates the β2AR–cAMP signaling pathway, upregulation of M3R, increases expression levels of PLCβ1 and IP3	Induces bronchoprotection tolerance	[107]
Nervous system						
Clenbuterol	HEK293 cells, mice	Cotransfected HEK293 cells with β2AR combined with an APP mutant (APPswe), APPswe/ PS1DE9 double-transgenic mice	Activated	Stimulates γ-secretase activity and amyloid plaque formation	Promotes the progression of Alzheimer’s disease	[129]
Clenbuterol	Male Wistar rats	Kainic acid (KA) induces inflammation and apoptosis in the hippocampus	Activated	Reduces inflammatory cytokine expression, reduced expression of iNOS, IDO, KMO and CD11b, coupled with increased BDNF and NGF expression	Antiinflammatory, neurotrophic, and neuroprotective effects	[131]
Clenbuterol	Mice	APP/PS1 mouse	Activated	Upregulation of postsynaptic density, synapsin 1 and synaptophysin, enhanced hippocampal neurogenesis, decreased cerebral amyloid plaques	Enhances neurogenesis and ameliorates memory deficits	[132]
ICI 118,551	Mice	3xTg-AD	Inhibited	Increases the level of Aβ and reducing the level of BDNF, increasing tau phosphorylation and accumulation	Exacerbates cognitive deficits	[133]
Salbutamol, clenbuterol, metaproterenol	SK-N-MC, primary cortical neurons in rats, iPSC neuron,mice	PD mice	Activated	Reduces the level of histone H3K27 acetylation (H3K27ac), reducing the accumulation of α-synuclein, and alleviate mitochondrial oxidative stress	Neuroprotective effect	[135]
Clenbuterol	Rat	LPS induces neuroinflammation	Activated	Inhibits the phosphorylation and degradation of IκBα and block the activation of NFκB	Reduces neuroinflammation	[142]
Immune system						
Isoproterenol, fenoterol	RAW264.7 cells	LPS induces inflammation	Activated	Stimulates the phosphorylation of protein kinase A (PKA) downregulates the expression of Toll-like receptor 4 (TLR4), thereby inhibiting the level of TNFα	Reduces inflammation	[150]
Formoterol	Bone marrow-derived macrophages (BMM)	LPS induces inflammation	Activated	Inhibits the secretion of TNFα and IL-8 and increase the secretion of IL-10	Prevents porcine macrophages from polarizing to the proinflammatory phenotype	[154]
Salmeterol	Murine microglial BV2 cells	LPS induces neurotoxicity	Activated	Activates cAMP–PKA–CREB as well as the PI3K and p38 MAPK signaling pathways	Neuroprotective effect	[156]
Terbutaline	C57BL/6 J mice, RAW 264.7 cells	Plantar skin wound model, LPS induces cellular inflammation	Activated	The upregulation of Trem1 was induced through the cAMP–PKA–CREB pathway	Amplifies the inflammatory response	[157]
Norepinephrine	DBA1/J mice	Collagen-induced arthritis (CIA)	Activated	Inhibits the differentiation and function of Th17 cells by activating the β2AR–PKA signaling pathway	Reduces the inflammatory response	[144]
Cancer						
ICI118,551	MIA PaCa-2, BxPC-3, BALB/c athymic nude mice	PanCa xenografts	Inhibited	Inhibits the cAMP–PKA and Ras signaling pathways	Inhibits the invasion and proliferation of pancreatic cancer	[160]
ICI118,551	MIA PaCa-2, BxPC-3, BALB/c athymic nude mice	PanCa xenografts	Inhibited	Inhibits Ras–Akt–NFκB pathway	Induces G1/S phase arrest and apoptosis in pancreatic cancer cells	[161]
Salbutamol	Mice	Tumor-bearing models	Active|Ated	Activates β2AR–ERK–EMT pathway	Promotes tumorigenesis of gastric cancer cells	[163]
Isoproterenol	MGC-803 and SGC-7901	/	Activated	Activates JAK–STAT3 signaling	Promotes epithelial–mesenchymal transition	[164]
Isoproterenol	HUVEC, MGC803, and HGC27	/	Activated	Activates Plexin-A1/VEGFR2 signals via VEGF secretion	Promotes tumor angiogenesis	[166]
ICI118,551	GES-1,BGC823, HGC27, MKN45, SGC7901, AGS, and MGC803	/	Inhibited	Inhibits the ERK1/2-JNK-MAPK pathway and transcription factors (such as NF-κB, AP-1, CREB, and STAT3)	Inhibits tumor proliferation, invasion, and metastasis	[168]
Propranolol and ICI 118,551	MDA-MB-231	/	Inhibited	Downregulating the ERK/COX-2 signaling pathway and inducing apoptosis	Inhibits cell viability	[174]
Isoproterenol	PC‐3 and LNCaP cells	/	Activated	Activates the sonic hedgehog-Gli1 signal	Drives prostate cancer progression	[176]
Isoproterenol	LNCaP and PC3 cells	/	Activated	Increases cAMP levels and ERK1/2 activation	Promotes the proliferation and cell migration of prostate cancer cells	[177]
Terbutaline	THP-1, HepG2 and SMMC-7721	/	Activated	Activates the β2-AR–cAMP–PKA–CREB and β2-AR–cAMP–IL-6/STAT3 pathways	Promotes the proliferation, migration and invasion of liver cancer cells	[182]
ICI-118,551, propranolol	CRC cell lines(SW1116, SW480, SW620, DLD1,HCT116, Colo205, and HT29)	/	Inhibited	Inhibits the EGFR-Akt–ERK1/2 pathway	Induces apoptosis of cancer cells	[181]
Atenolol, propranolol, ICI-118,551	ccRCC primary tumors, 786-O cells, and HUVECs	/	Inhibited	Activates apoptosis, inhibit the gene and protein expressions of HIF-2α, CAIX and VEGF, and partially affect the nucleation of HIF-2α and NF B/p65	Inhibits the proliferation of cancer cells, tumor angiogenesis, and inflammation	[183]
Propranolol and ICI-118,551	ccRCC primary tumors and the 786-O cell line, tumor-bearing mouse	/	Inhibited	Reduces inflammation and oxidative stress	Antitumor effect	[183]
Isoprenaline, formoterol	NB cells (SK-N-AS, SK-N-SH, SHEP, SK-N-BE2, SK-N-BE2c)	/	Activated	triggers autophagy through CREB‑mediated ULK1 upregulation	Promotes the growth of neuroblastoma	[184]
Isoprenaline	A549 cells	/	Activated	Upregulates the expressions of MMP-2/9 and VEGF through the ERK1/2/CREB signaling pathway	Promotes tumor progression	[185]
Albutamol/albuterol (SABA)	Human airway smooth muscle	Asthma / acute bronchospasm	Activated	Gαs-biased agonism rapidly activates Gαs–cAMP–PKA in airway smooth muscle, with comparatively low β-arrestin-mediated desensitization at clinical short-term doses	Rescue bronchodilation; clinically used as a SABA for acute relief of asthma and COPD	[69, 117, 186]
Salmeterol /formoterol (LABAs)	Human airway smooth muscle, bronchial epithelium	Persistent asthma/COPD (chronic maintenance)	Activated (chronic)	Sustained Gαs/cAMP/PKA activation with substantial β-arrestin recruitment, GRK-driven desensitization, PDE4D induction, and CREB-dependent miRNA let-7f upregulation	Long-acting bronchodilation; chronic monotherapy associated with tachyphylaxis and asthma-related mortality (FDA black-box warning), motivating Gαs-biased successors	[95, 108, 109, 116, 117, 119]
Carvedilol	Failing human/rodent myocardium	Heart failure with reduced ejection fraction (HFrEF), post-MI	Inhibited (Gαs)/β-arrestin-biased agonism	Inverse agonist for β2AR-Gαs/cAMP while activating β-arrestin-dependent ERK1/2; cryo-EM-defined β2AR conformation; positive cooperativity with allosteric modulator Cmpd-6FA	Improves survival in HFrEF; β-arrestin-biased profile proposed to underlie superior mortality benefit vs. non-biased β-blockers (clinical advantage supported but mechanism debated)	[8, 97, 98, 112]
Propranolol (nonselective β-blocker)	Cardiovascular system, peripheral tissues	Hypertension, arrhythmia, post-MI; oncology adjunct	Inhibited	Nonselective β1/β2 antagonist; in oncology blocks β2AR-Gαs–cAMP and Ras–ERK signaling and reduces VEGF/MMP	Blood pressure/arrhythmia control; emerging adjunctive antitumor activity (e.g., in TNBC, ccRCC, pancreatic cancer)	[160, 161, 173, 182, 183]
Bisoprolol (cardio-selective β1-blocker)	Failing human myocardium	Heart failure (HFrEF)	Indirect (β1-selective antagonism, β2AR signaling preserved)	Selective β1-blockade with negligible β2AR antagonism; spares β2AR cardioprotective Gαi-PI3K–Akt signaling in failing hearts	Improves survival in HFrEF; rationale for combination with future β2AR-biased agonists that enhance β-arrestin-dependent cardioprotection	[70, 94]

Despite the conceptual promise of biased ligands, their clinical translation has faced significant hurdles. A central challenge is the distinction between system bias and ligand bias: a ligand that appears biased in recombinant cell systems with defined receptor–effector stoichiometries may display different or even opposite bias profiles in native tissues where receptor density, GRK isoform expression, and β-arrestin levels vary. Consequently, signaling outcomes observed in simplified experimental systems may not accurately predict pharmacological responses in physiological settings [155]. Furthermore, the TRV027 clinical experience at the angiotensin II type-1 receptor (AT1R) is invoked here purely as a cross-receptor functional analogy not as a β2AR ligand and illustrates the broader translational challenges of biased GPCR pharmacology that are likely to be encountered with β2AR biased ligands as well. Although TRV027 showed promising β-arrestin-biased signaling and cardioprotective effects in preclinical models, the BLAST-AHF phase IIb trial failed to demonstrate improvement in clinical outcomes in patients with acute heart failure [96]. This outcome highlights the difficulty of translating pathway-selective signaling observed in controlled experimental systems into therapeutic benefit in complex human disease. Additional barriers include the absence of validated in vivo biomarkers for pathway-selective engagement, species differences in β-adrenergic receptor pharmacology and GRK expression, and the lack of regulatory frameworks specifically designed for evaluating pathway-selective drugs. These challenges underscore the need for systematic characterization of bias factors across multiple physiologically relevant cell types before advancing biased ligands into clinical development.

Moving forward, elucidating the molecular mechanisms underlying β2AR-biased signaling is essential for developing next-generation ligands with enhanced selectivity and improved safety profiles. Integration of structural biology advances with computational pharmacology will enable rational drug design and refined receptor–ligand interaction models. These efforts hold promise for translating β2AR-targeted therapeutics into precision treatments tailored to specific disease endotypes, ultimately improving clinical outcomes across diverse pathological conditions.

### Structural basis of β2AR biased agonism

The structural basis of biased agonism at β2AR has been illuminated by several key studies. Liu et al. [28] placed site-specific 19F-NMR labels on β2AR and demonstrated that agonist binding primarily shifts the equilibrium of helix VI toward the G protein-specific active state, while β-arrestin-biased ligands predominantly impact helix VII conformational states. This provides a structural explanation for why different ligands produce different signaling outcomes. Cryo-EM structures of the β2AR–Gs complex [192] revealed the outward movement of transmembrane helix 6 upon Gs engagement. Rather than a single active state, β2AR exists in a dynamic equilibrium of multiple conformations (conformational ensembles), and biased ligands shift this ensemble to favor particular states that preferentially couple to specific effectors. The intermediate conformational states detected by 19F-NMR still need to be resolved at atomic resolution by cryo-EM. More recently, cryo-EM studies of β2AR in complex with different transducers have revealed that the G protein-bound and β-arrestin-bound conformations differ primarily in the orientation of transmembrane helices V and VI and the conformation of intracellular loop 2, providing atomic-level insight into how biased ligands achieve pathway selectivity [193].

### Quantifying biased agonism: the operational model framework

Quantification of biased agonism relies on the Black–Leff operational model, which describes agonism through two key parameters: KA (receptor affinity) and τ (transducer ratio/efficacy). Kenakin et al. [30] developed the transduction coefficient log(τ/KA), which combines affinity and efficacy into a single parameter for comparing agonists. Bias factors are calculated as ΔΔlog(τ/KA) values by first normalizing to a reference agonist (Δlog(τ/KA)) in each pathway, then comparing across pathways. A statistically significant ΔΔlog(τ/KA) ≠ 0 indicates bias. Bias plots (equimolar comparison plots) serve as a complementary visualization tool. However, system-dependent variations in receptor density and effector availability mean that bias factors measured in recombinant systems may not translate directly to native tissues, necessitating validation in physiologically relevant models.

### System-dependent modifiers of bias

Several system-dependent factors modulate the observed bias at β2AR. Receptor expression stoichiometry differs between cell types, influencing receptor reserve and potentially masking or amplifying ligand bias. GRK isoform availability is another critical modifier: GRK2/3 versus GRK5/6 phosphorylate different residues on the receptor C-terminus, generating distinct “phosphorylation barcodes” that dictate downstream β-arrestin conformation and function. Kawakami et al. [55] demonstrated that heterotrimeric Gq proteins act as a switch for GRK5/6 selectivity underlying β-arrestin transducer bias at AT1R and other Gq-coupled receptors (e.g., 5-HT2A, NTS1). This Gq-dependent switching mechanism does not generalize to Gs-coupled receptors such as β2AR; for β2AR, GRK5/6 selectivity is instead governed by ligand-induced receptor conformations and the kinetics of direct GRK recruitment, with Gαs–GRK2/3 axis providing complementary phosphorylation of distinct C-terminal residues [27, 28, 54]. Additionally, compartmentalized cAMP signaling from plasma membrane versus endosomal β2AR generates spatially distinct cAMP pools with different downstream transcriptional outcomes (see Compartmentalized β2AR Signaling section above).

### Pharmacogenomics of β2AR: implications for biased signaling and precision therapy

Three major coding polymorphisms in the β2AR gene (ADRB2) have well-characterized functional consequences relevant to biased signaling: (1) Arg16Gly (rs1042713), located in the extracellular N-terminus, modulates agonist-induced receptor downregulation, with the Gly16 variant displaying enhanced downregulation that may alter the kinetics of both Gαs- and β-arrestin-mediated signaling [113]; (2) Gln27Glu (rs1042714), also in the N-terminal region, where the Glu27 variant is more resistant to agonist-induced downregulation, potentially sustaining Gs-biased signaling under chronic catecholamine exposure; and (3) Thr164Ile (rs1800888), located in transmembrane domain IV, which significantly reduces receptor coupling efficiency to Gs and impairs agonist-stimulated adenylyl cyclase activation [69]. These polymorphisms intersect with ligand-directed bias: a biased ligand designed to preferentially engage β-arrestin signaling may show differential efficacy in patients carrying variants that alter Gs coupling efficiency. Pharmacogenomic stratification could therefore be essential for clinical development of β2AR biased therapeutics.

## Conclusion and future prospects

β2AR can selectively activate distinct downstream pathways through biased signaling. Its signal output is regulated by multiple factors such as ligand properties, receptor modifications, and the cellular environment. This adaptability highlights β2AR’s essential function in regulating several pathophysiological processes in respiratory, neurological, cardiovascular, and immune-related illnesses. The strategic targeting of biased ligands associated with β2AR can effectively modulate the activation of G proteins or the inhibition of the β-arrestin pathway, thereby facilitating the development of safer therapeutic interventions for diseases such as asthma and heart failure. Future research should focus on clarifying the connection between β2AR conformational dynamics and signaling bias, identifying the molecular factors responsible for ligand-induced structural transitions, and examining how these findings could be applied to precision-targeted medical treatments. By developing pathway-specific ligands and optimizing the design of signal preference, it is expected to maximize therapeutic effects, minimize side effects, and achieve individualized treatment, opening up new avenues for GPCR-based therapy.

Several specific unresolved questions warrant attention. First, although β2AR structures in complex with Gαs have been resolved [192] and initial β-arrestin engagement data obtained, the conformational ensembles stabilized by partial agonists, allosteric modulators, and receptor polymorphic variants remain structurally uncharacterized; the intermediate conformational states detected by 19F-NMR need to be resolved at atomic resolution by cryo-EM. Second, ICL1-9 has demonstrated β-arrestin-biased agonism and calcium-independent enhancement of cardiomyocyte contractility in vitro, but in vivo validation is lacking; preclinical pharmacokinetic, efficacy, and safety studies in animal models of heart failure and ischemia/reperfusion injury are essential before clinical translation. Third, disentangling system bias from ligand bias requires systematic measurement of bias factors across multiple cell types with varying GRK isoform and β-arrestin expression profiles. Fourth, key translation barriers include species differences in β2AR pharmacology and GRK expression, the absence of validated in vivo biomarkers for biased signaling, and the current lack of regulatory frameworks specifically designed for pathway-selective drugs.

Following receptor activation, G protein-coupled receptor kinases (GRKs) phosphorylate intracellular residues on β2AR, creating binding sites for β-arrestin. β-arrestin recruitment promotes receptor desensitization by sterically preventing further G-protein coupling and facilitates receptor internalization through clathrin-mediated endocytosis. In addition to its role in receptor trafficking, β-arrestin functions as a signaling scaffold that organizes MAPK signaling complexes and activates ERK1/2, p38, and JNK pathways, regulating inflammatory signaling, gene transcription, and cell survival. Internalized β2AR can continue signaling from endosomal compartments, generating spatially restricted cAMP pools and activating RAF–MEK–ERK signaling pathways that regulate nuclear transcription programs. This spatially restricted signaling represents an example of compartmentalized or location-biased signaling.Together, these signaling branches illustrate how β2AR functions as a multifunctional signaling hub in which ligand-dependent receptor conformations, transducer coupling, and subcellular localization collectively determine signaling bias and downstream cellular outcomes. Image created with Biorender.com.

## Data Availability

No datasets were generated or analyzed during the current study.

## Abbreviations

AC: Adenylyl cyclase
Ach: Acetylcholine
AD: Alzheimer’s disease
AKAPs: A-kinase anchoring proteins
AKR1B1: Aldehyde-ketone reductase family 1 member B1
APP/PS1: Amyloid precursor protein/presenilin 1
ASMCs: Airway smooth muscle cells
ATP: Adenosine triphosphate
APP: Amyloid precursor protein
Aβ: Amyloid-beta protein
BDNF: Brain-derived neurotrophic factor
BKCa: Large-conductance calcium-activated potassium
BMM: Bone marrow-derived macrophages
cAMP: Cyclic adenosine monophosphate
cAMP-PKA: Cyclic adenosine monophosphate-protein kinase A
cPLA2: Cytoplasmic phospholipase A2
c-Src: Proto-oncogene tyrosine-protein kinase Src
CaN: Calcineurin
CAIX: Carbonic anhydrase IX
CcRCC: Clear cell renal cell carcinoma
CCR2: C–C motif chemokine receptor 2
CFTR: Cystic fibrosis transmembrane conductance regulator
CIA: Collagen-induced arthritis
COPD: Chronic obstructive pulmonary disease
COX-2: Cyclooxygenase-2
CRC: Colorectal cancer
CREB: Cyclic adenosine monophosphate response element binding protein
Cx43: Gap junction protein 43
DCs: Dendritic cells
eNOS: Endothelial nitric oxide synthase
ENaC: Epithelial sodium channel
Εpkc: Epsilon Protein kinase C
EPSCs: Excitatory postsynaptic current
ERK1/2: Extracellular signal-regulated kinases 1 and 2
ERK/p38-ATF: Extracellular signal-regulated protein kinase/p38-activating transcription factor
GluR1: Glutamate receptor subunit 1
GM-CSF: Granulocyte–macrophage colony-stimulating factor
GPCR: G protein-coupled receptor
GRK2: G protein-coupled receptor kinase 2
HASM: Human airway smooth muscle (cells)
HF: Heart failure
HIF-2α: Hypoxia-inducible factor-2α
HPV: Hypoxic pulmonary vasoconstriction
HSCs: Hepatic stellate cells
HUVEC: Human umbilical vein endothelial cells
H3K27ac: H3K27 acetylation
ICAM-1: Intercellular adhesion molecule-1
ICL1-9: Intracellular loop1-9
IDO: Indoleamine-2,3-dioxygenase
IH: Intermittent hypoxia
IL-6: Interleukin-6
iPSC: Induced pluripotent stem cell
IP3: Inositol 1,4,5-trisphosphate
I/R: |Ischemia/reperfusion
ISO: Isoproterenol
JAK-STAT3: Janus kinase-signal transducer and activator of transcription 3
JNK: C-Jun N-terminal kinase
KA: Kainic acid
KMO: Kynurenine 3-monooxygenase
LABAs: Long-acting β2-adrenergic receptor agonists
LAMAs: Long-acting muscarinic antagonists
LNCaP: Lymph node carcinoma of the prostate
LPS: Lipopolysaccharide
LTP: Long-term potentiation
LX-2: Lieming Xu-2 cell
MAPK: Mitogen-activated protein kinase
MMP-2: Matrix metalloproteinase 2
MS: Multiple sclerosis
M3R: M3 receptor
NB: Neuroblastoma
NE: Norepinephrine
NF-κB: Nuclear factor-kappa B
NGF: Nerve growth factor
NO: Nitric oxide
PanCa: Pancreatic cancer
PD: Parkinson’s disease
PDE4D: Phosphodiesterase 4D
PDE4D5: Phosphodiesterase 4D5
PIP2: Phosphatidylinositol-4,5-bisphosphate
PIP3: Phosphatidylinositol-3,4,5-trisphosphate
PI3K-Akt: Phosphatidylinositol 3-kinase-serine/threonine kinase
PKC: Protein kinase C
PLCβ1: Phospholipase Cβ1
PPAR-γ: Peroxisome proliferator-activated receptor γ
RORC: RAR-related orphan receptor C
SABAs: Short-acting β2-adrenergic receptor agonists
SNCA: α-Synuclein
STAT3: Signal transducer and activator of transcription 3
TAMs: Tumor-associated macrophages
TCR: T-cell receptor
TLR4: Toll-like receptor 4
TNBC: Triple-negative breast cancer
Tregs: Regulatory T cells
ULK1: UNC-51-like kinase 1
VCAM: Vascular cell adhesion molecule
VDC: Voltage-dependent calcium channel
VEGF: Vascular endothelial growth factor
VEGFA: Vascular endothelial growth factor A
VEGF/FGF2: Vascular endothelial growth factor/fibroblast growth factor 2
16-HBE: Human bronchial epithelial cells
β2AR: β2-Adrenergic receptor
ZINT: Zinterol (β2-adrenergic receptor agonist)
